# Methylation of HBP1 by PRMT1 promotes tumor progression by regulating actin cytoskeleton remodeling

**DOI:** 10.1038/s41389-022-00421-7

**Published:** 2022-08-08

**Authors:** Jiyin Wang, Ruixiang Yang, Yuning Cheng, Yue Zhou, Tongjia Zhang, Shujie Wang, Hui Li, Wei Jiang, Xiaowei Zhang

**Affiliations:** Department of Biochemistry and Biophysics, School of Basic Medical Sciences, Beijing Key Laboratory of Protein Posttranslational Modifications and Cell Function, Peking University Health Science Center, Beijing, 100191 P. R. China

**Keywords:** Cancer, Molecular biology

## Abstract

HBP1 is a sequence-specific transcription factor which generally considered as a crucial growth inhibitor. Posttranslational modification of HBP1 is vital for its function. In this study, we demonstrate that HBP1 is methylated at R378 by PRMT1, which decreases HBP1 protein stability by promoting its ubiquitination and proteasome-mediated degradation. PRMT1-mediated methylation of HBP1 alleviates the repressive effects of HBP1 on tumor metastasis and growth. *GSN* is identified as a novel target gene of HBP1. Methylation of HBP1 promotes actin cytoskeleton remodeling, glycolysis and tumor progression by downregulating GSN (a vital actin-binding protein) levels. The methylated HBP1-GSN axis is associated with the clinical outcomes of cancer patients. This investigation elucidates the mechanism of how methylated HBP1 facilitates actin cytoskeleton remodeling, thus attenuates its tumor-suppressive function and promotes tumor progression. Targeting methylated HBP1-GSN axis may provide a therapeutic strategy for cancer.

## Introduction

Transcription factor HBP1 is a member of the sequence-specific high mobility group (HMG) protein family. HBP1 plays a vital role in cell metabolism and cell cycle progression by regulating the expression of a number of important cell cycle regulators. Several micro-RNAs can epigenetically regulate the expression of HBP1 in cancer cells, such as miR-17-5p and miR-21, which can improve cell proliferation, invasion and migration by decreasing the expression of HBP1 [1, 2]. As a dual transcription factor, HBP1 can repress the transcription of certain target genes, such as *N-MYC, DNMT1, C-MYC, and EZH2*, via direct binding to the high-affinity sites [3–6]. However, HBP1 can also transcriptionally activate several genes, including *p16, PIM-1, p21, MPO*, and *histone H1* [6–10]. Whether HBP1 acts as a repressor or activator depends on specific post-translational modifications (PTMs) and promoter DNA sequences. We have previously reported that HBP1 is a substrate for PIM-1 kinase during oxidative stress [8]. PIM-1-mediated phosphorylation of HBP1 increases its protein stability and, in turn, induces cell growth arrest by activating *PIM-1* gene expression. Moreover, HBP1 can be phosphorylated by p38 MAPK and participates in Ras-p38 MAPK-induced premature senescence [11]. A specific p300/CBP acetylation site (Lys-419) has been identified in the HBP1 protein [12]. The acetylation of HBP1 is required for enhancing p16 transcription and G1 cell cycle arrest. HBP1 is also a target of E3 ubiquitin ligase MDM2. MDM2 can ubiquitinate HBP1, leading to proteasomal degradation. This can thus attenuate HBP1-mediated transcriptional repression of *EZH2* and *DNMT1*, resulting in histone hypermethylation, global DNA hypermethylation, and ultimately genomic instability [13]. These data collectively indicate that HBP1 modulation is a complex process and PTMs play crucial roles in the subsequent biological consequences, as HBP1 abrogation disrupts cell metabolism and promotes tumorigenesis. Similar to phosphorylation, acetylation, and ubiquitination, arginine methylation is also a common PTM. Currently, there is no report regarding the biological significance of methylated HBP1. The objectives of this study were to investigate if HBP1 can be methylated by protein arginine methyltransferase (PRMT) and if methylated HBP1 can influence downstream transcriptional activity and relevant biological functions.

Protein methylation at arginine residues is mainly catalyzed by the PRMT protein family. PRMTs are involved in many biological processes including signal transduction, RNA processing, DNA repair, DNA replication, and gene transcription [14–17]. The PRMT family includes three groups categorized by catalytic activity. TypeIenzymes (PRMT1-4, PRMT6, and PRMT8) can generate monomethylation and asymmetric dimethylarginine residues, typeIIenzymes (PRMT5 and PRMT9) catalyze monomethylation and create symmetric dimethylarginine residues, and type III (PRMT7) only catalyzes monomethylation [15]. PRMT1 is known to be a typeIenzyme and performs 85% of total PRMT activity in mammalian cells. It can methylate both histone and non-histone proteins, such as H4, FOXO1, C/EBPα, EGFR, Twist1, and others [18–22]. However, it remains unknown whether HBP1 is methylated by PRMTs.

GSN (Gelsolin) is an abundant actin-binding protein that mediates the rate of actin-based cell migration. It regulates the actin cytoskeleton by severing, capping, and nucleating actin filaments, as well as sequestering actin monomers [23]. Yet, the role of GSN in different tumors is controversial. In breast cancer and hepatocellular carcinoma (HCC), GSN promotes cell proliferation and metastasis [24, 25]. However, overexpression of GSN in bladder cancer and glioblastoma (GBM) cells leads to a loss of tumorigenicity [26, 27]. The function of *GSN* as both a growth inhibitor gene and oncogene depends on the specific carcinoma type through regulation of actin cytoskeleton remodeling and cell motility. Both roles mark GSN as a prospective target for novel cancer therapies.

In the present study, we demonstrate that HBP1 can be methylated by PRMT1 at R378, which alleviates HBP1-mediated repression of tumor metastasis and growth through regulation of GSN expression. GSN is identified as a novel target of HBP1. Methylated HBP1 can decrease protein stability by promoting its ubiquitination and proteasome-mediated degradation, thereby reducing GSN expression and promoting actin cytoskeleton remodeling and tumor progression. The PRMT1-HBP1-GSN axis is critical for regulating cytoskeleton remodeling and tumorigenesis, and targeting this axis may provide a new therapeutic strategy for treating various cancers.

## Results

### HBP1 is methylated by PRMT1 at arginine 378

**Fig. 1:**
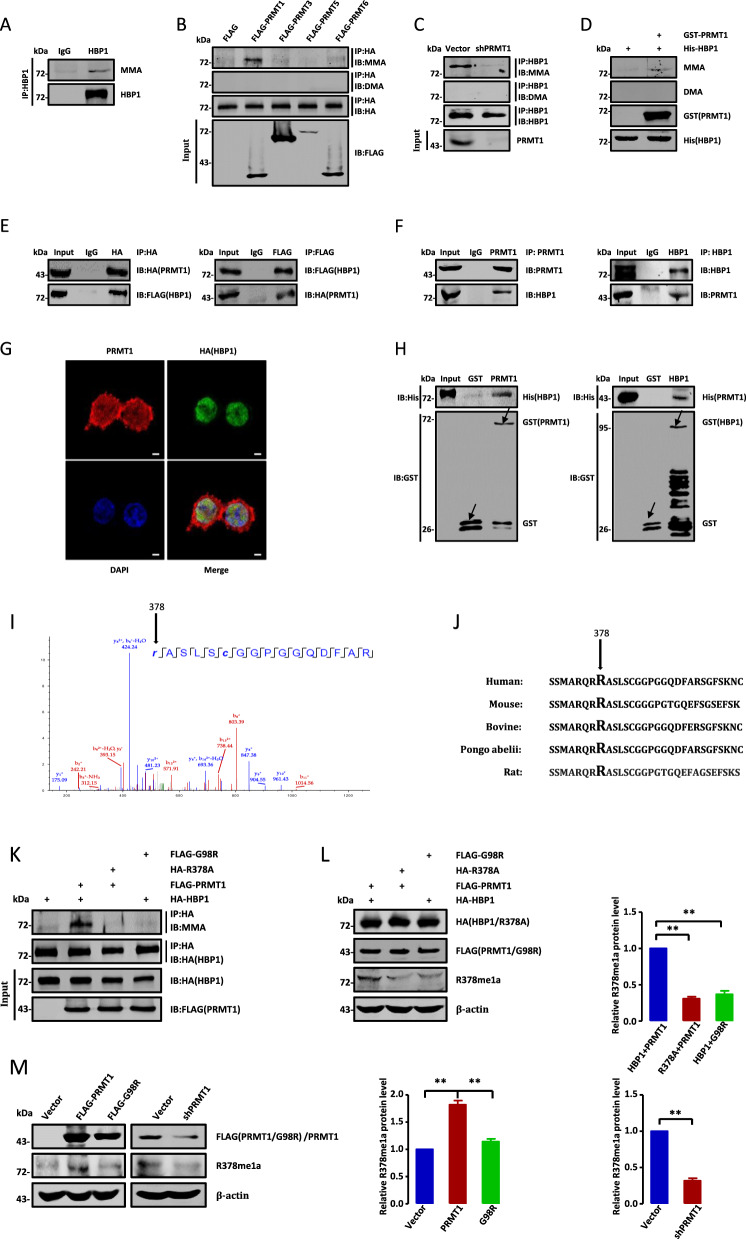
HBP1 is methylated by PRMT1 at arginine 378. **A** Endogenous HBP1 undergoes MMA modification. Endogenous HBP1 immunoprecipitates from HeLa cells were immunoblotted with anti-HBP1 and anti-MMA antibodies. **B** HBP1 is methylated by PRMT1 in vivo. HA-HBP1 with FLAG-PRMT1, 3, 5, 6 individually were co-transfected into HEK293T cells. Then total cell lysates were immunoprecipitated with anti-HA antibody and detected by western blotting. **C** Knock down PRMT1 decreased HBP1 methylation. Endogenous HBP1 immunoprecipitates in HeLa cells with PRMT1 knock down and then blotted with anti-MMA antibody and anti-DMA antibody. **D** HBP1 is methylated by PRMT1 in vitro. Purified His-HBP1 was incubated with or without GST-PRMT1 in 60 μL of HMT buffer at 37 °C for 2 h and followed by western blotting with His, GST, MMA or DMA antibodies. **E**, **F** PRMT1 interacts with HBP1 in vivo. **E** HEK293T cells were co-transfected with HA-HBP1 and FLAG-PRMT1. The Co-IP assay was carried out by using anti-HA/FLAG antibody and followed by western blotting with anti-FLAG/HA antibody. **F** Endogenous HBP1 or PRMT1 immunoprecipitates from HeLa cells were immunoblotted with anti-HBP1 or anti-PRMT1 antibodies. **G** PRMT1 co-localizes with HBP1 in nucleus. HEK293T cells were transfected with HA-HBP1 and stained with PRMT1 antibody (red) and anti-HA antibody (green). DAPI (blue), nucleus. Scale bar, 5 µm. **H** PRMT1 interacts with HBP1 in vitro. GST pull-down assays were performed using His-HBP1 /His-PRMT1 with GST or GST-PRMT1/GST-HBP1 followed by immunoblotting with anti-His antibody. **I**–**M** PRMT1 methylates HBP1 at arginine 378. **I** HBP1 methylation is analyzed by mass spectrometry. **J** Evolutionary conservation of the HBP1 R378 residue in other species. **K** HEK293T cells were co-transfected with HA-HBP1/R378A with FLAG-PRMT1/G98R and subjected to immunoprecipitation followed by western blotting. **L**, **M** HBP1 methylation was verified by polyclonal antibody specific for HBP1 R378me1a.

To ascertain whether HBP1 arginine residues are methylated in vivo, we performed co-immunoprecipitation (co-IP) experiments in HeLa cells with an HBP1 antibody and detected monomethylated arginine (MMA) using an additional antibody. As shown in Fig. 1A, endogenous HBP1 underwent MMA modification. To identify the specific methylating enzyme(s), we co-transfected HEK293T cells with HA-HBP1 and FLAG-PRMT1, 3, 5, or 6 individually. IP analysis indicated that HBP1 was monomethylated by PRMT1 (Fig. 1B). Furthermore, we knocked down PRMT1 expression by shRNA in HeLa cells and performed IP experiment with an HBP1 antibody, then detected the level of methylated-HBP1. IP analysis indicated that monomethylated-HBP1 was decreased by PRMT1 knocked down. (Fig. 1C). To validate the results, we performed in vitro methylation assays. Recombinant His-HBP1 protein was monomethylated, but not asymmetrically dimethylated (DMA), following addition of PRMT1 (Fig. 1D). Taken together, the results demonstrate that HBP1 can be methylated by PRMT1 both in vivo and in vitro. Next, we investigated whether HBP1 combines with PRMT1. We ectopically expressed HA-PRMT1 and FLAG-HBP1 in HEK293T cells and then performed co-IPs with an anti-HA or anti-FLAG antibody. The results show that exogenous HBP1 can bind to exogenous PRMT1 in vivo (Fig. 1E). An endogenous interaction between PRMT1 and HBP1 was validated in HeLa cells (Fig. 1F). We also employed immunofluorescence staining assays to test the PRMT1/HBP1 interaction. As shown in Fig. 1G, PRMT1 co-localized with HBP1 in the nucleus. Finally, we performed GST pull-down assays to clarify the direct interaction between PRMT1 and HBP1, which further demonstrated the PRMT1/HBP1 interaction in vitro (Fig. 1H).

We then performed mass spectrometry analysis to identify PRMT1-specific methylation sites in HBP1. FLAG-HBP1 was ectopically expressed alone or co-expressed with PRMT1 in HEK293T cells, followed by affinity purification using protein A-Sepharose and resolved by SDS-PAGE (Fig. S1). This analysis revealed that HBP1 was monomethylated by PRMT1 at R378 (Fig. 1I). Notably, HBP1 R378 is highly evolutionarily conserved across species, such as human, mouse, and bovine, etc. (Fig. 1J), suggesting that methylation of that specific amino acid may be important for HBP1 function. To further confirm R378 methylation by PRMT1, we designed two mutants: R378A (methylation-deficient HBP1 mutant) and G98R (methyltransferase dead PRMT1 mutant) [28]. We then co-transfected HEK293T cells with HA-HBP1 or R378A mutant together with FLAG-PRMT1 or G98R mutant. As shown in Fig. 1K, HBP1 methylation was only observed in the cells co-transfected with wild-type HBP1 and wild-type PRMT1, indicating that exogenous HBP1 is specifically methylated by PRMT1 at R378. Additionally, we generated a polyclonal antibody specific for HBP1 protein methylated at R378, which was designated as R378me1a. HeLa cells were transiently transfected with HBP1 + PRMT1, R378A + PRMT1, or HBP1 + G98R. HBP1 methylation levels were then measured using western blotting analysis with the R378me1a antibody. As shown in Fig. 1L, only wild-type HBP1 was methylated in the presence of PRMT1 expression, indicating monomethylation of R378 in vivo. Furthermore, we transfected FLAG-PRMT1, G98R or PRMT1shRNA into HeLa cells and measured endogenous HBP1 methylation level using R378me1a antibody. As shown in Fig. 1M, PRMT1 overexpression increased endogenous HBP1 methylation level, while PRMT1 knockdown decreased HBP1 methylation level. We therefore conclude that exogenous or endogenous HBP1 can be monomethylated by PRMT1 at R378 in vivo.

### HBP1 methylation by PRMT1 decreases HBP1 stability by promoting MDM2-mediated ubiquitination

**Fig. 2:**
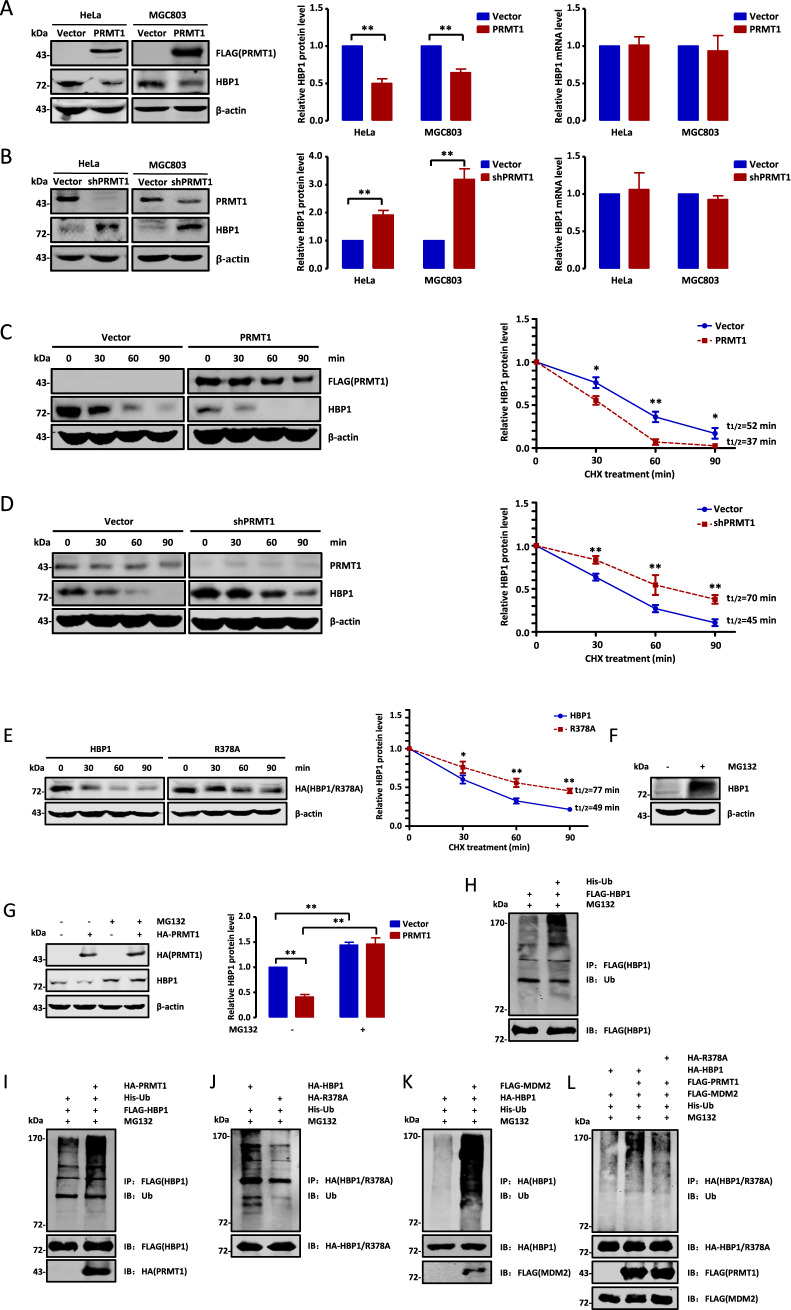
HBP1 methylation by PRMT1 decreases HBP1 stability by promoting its ubiquitination. **A**, **B** PRMT1 reduces HBP1 protein levels. Western blot analysis of HBP1 in HeLa and MGC803 cells transfected with empty vector or FLAG-PRMT1 (**A**, left and middle panels)/shPRMT1 (**B**, left and middle panels). mRNA expression of endogenous HBP1 measured by real-time PCR in HeLa and MGC803 cells transfected with empty vector or FLAG-PRMT1 (**A**, right panels)/shPRMT1 (**B**, right panels) (*n* = 3, Student’s *t*-test). **C**, **D** PRMT1 decreases HBP1 protein stability. Western blot of endogenous HBP1 expression in empty vector- or FLAG-PRMT1(**C**)/shPRMT1(**D**)-transfected HeLa cells when treated with CHX for 0, 30, 60, or 90 min (*n* = 3, Student’s *t*-test). **E** PRMT1-mediated HBP1 methylation decreases HBP1 protein stability. The stable expression of wild-type HBP1 and mutant R378A were detected by western blotting in transfected HeLa cells when treated with CHX for 0, 30, 60, or 90 min (*n* = 3, Student’s *t*-test). The half-life of HBP1 protein t1/2 measurements were performed as described previously [11]. **F** HBP1 protein level is elevated in the presence of MG132. HeLa cells were treated with MG132 for 6 h, the protein level of HBP1 was measured by western blotting. **G** PRMT1 does not decrease HBP1 protein level in the presence of MG132. HeLa cells were transfected with empty vector or FLAG-PRMT1 and incubated with or without MG132 for another 6 h. The protein level of HBP1 was detected by western blotting. **H**–**L** PRMT1-mediated methylation of HBP1 promotes its ubiquitination. **H** HEK293T cells were co-transfected FLAG-HBP1 with or without His-ubiquitin and then immunoprecipitated with anti-FLAG antibody. The HBP1 ubiquitination was measured by western blotting with anti-multi ubiquitin antibody. **I** HEK293T cells were co-transfected FLAG-HBP1, His-ubiquitin with or without HA-PRMT1 and then immunoprecipitated with anti-FLAG antibody. The HBP1 ubiquitination was measured by western blotting with anti-multi ubiquitin antibody. **J** HEK293T cells were co-transfected His-ubiquitin with HA-HBP1 or HA-R378A and then immunoprecipitated with anti-HA antibody. The HBP1 ubiquitination was measured by western blotting. **K** HEK293T cells were co-transfected HA-HBP1, His-ubiquitin with or without FLAG-MDM2 and then immunoprecipitated with anti-HA antibody. The HBP1 ubiquitination was measured by western blotting with anti-multi ubiquitin antibody. **L** HEK293T cells were co-transfected His-ubiquitin, MDM2, HA-HBP1 or HA-R378A with or without FLAG-PRMT1 and then immunoprecipitated with anti-HA antibody. The HBP1 ubiquitination was measured by western blotting. Date were the mean ± SD. *p < 0.05, **p < 0.01.

To clarify if PRMT1-mediated HBP1 methylation could regulate HBP1 expression, we first tested the effect of PRMT1 overexpression on HBP1 protein and mRNA levels by western blotting and real-time PCR, respectively. As shown in Fig. 2A, PRMT1 overexpression decreased HBP1 protein levels in both HeLa and MGC803 cells, but had no effect on HBP1 mRNA levels. To further confirm PRMT1 function, we knocked down PRMT1 expression in HeLa and MGC803 cells using PRMT1shRNA. Efficient shRNA-mediated PRMT1 knockdown promoted HBP1 protein levels, but caused little change to HBP1 mRNA levels (Fig. 2B). Thus, these results suggest that PRMT1 represses HBP1 protein expression at a post-transcriptional level. To investigate how PRMT1 regulates HBP1, we tested the stability of HBP1 using protein half-life experiments. As shown in Fig. 2C, D, PRMT1 overexpression clearly decreased the half-life of HBP1 from 52 min to 37 min (Fig. 2C), whereas knocking down PRMT1 significantly increased the half-life of HBP1 from 45 min to 70 min (Fig. 2D). To further determine whether HBP1 degradation can be attributed to methylation of R378, we expressed wild-type HBP1 or R378A mutant in HeLa cells. Notably, wild-type HBP1 protein levels decayed at a faster rate than R378A mutant HBP1 protein in cells incubated with cycloheximide. Comparing with wild-type HBP1, R378A mutant increased the half-life of HBP1 from 49 min to 77 min (Fig. 2E). Thus, we conclude that PRMT1-mediated methylation of HBP1 can decrease HBP1 protein levels by reducing its protein stability.

We next sought to elucidate the mechanism by which methylation of HBP1 decreases its protein levels. Because proteasome-dependent degradation of proteins is promoted by ubiquitination, we hypothesized that methylation could decrease HBP1 stability by promoting its ubiquitination. We incubated HeLa cells with MG132, a proteasomal degradation inhibitor, and assessed HBP1 protein levels by western blotting. Indeed, MG132 treatment resulted in a marked increase in HBP1 levels, and the accumulation was not decreased by PRMT1 overexpression, indicating that PRMT1-mediated methylation decreased HBP1 stability in a proteasome-dependent manner (Fig. 2F, G). To determine whether methylation of HBP1 could promote its ubiquitination and degradation, we used HEK293T cells expressing FLAG-HBP1 with or without His-ubiquitin, then detected HBP1 ubiquitination using an anti-multi ubiquitin antibody. As shown in Fig. 2H, HBP1 protein could be ubiquitinated in vivo. In addition, HBP1 ubiquitination was increased in HEK293T cells transfected with HA-PRMT1 (Fig. 2I). Moreover, the ubiquitination level of wild-type HBP1 was markedly higher than that of R378A (Fig. 2J), suggesting that HBP1 methylation at R378 by PRMT1 promotes its ubiquitination. We previously reported that HBP1 is ubiquitinated by ubiquitin E3 ligase MDM2 [13]. We then tested if HBP1 methylation at R378 promotes MDM2-mediated ubiquitination. As shown in Fig. 2K, L, HBP1 was ubiquitinated by MDM2 and PRMT1 increased wild-type HBP1 ubiquitination mediated by MDM2, while had no effect on R378A. Altogether, our results indicate that HBP1 methylation at R378 by PRMT1 promotes MDM2-mediated ubiquitination, therefore decreases HBP1 protein stability.

### HBP1 methylation alleviates HBP1-mediated suppression of metastasis and growth of tumor cells

**Fig. 3:**
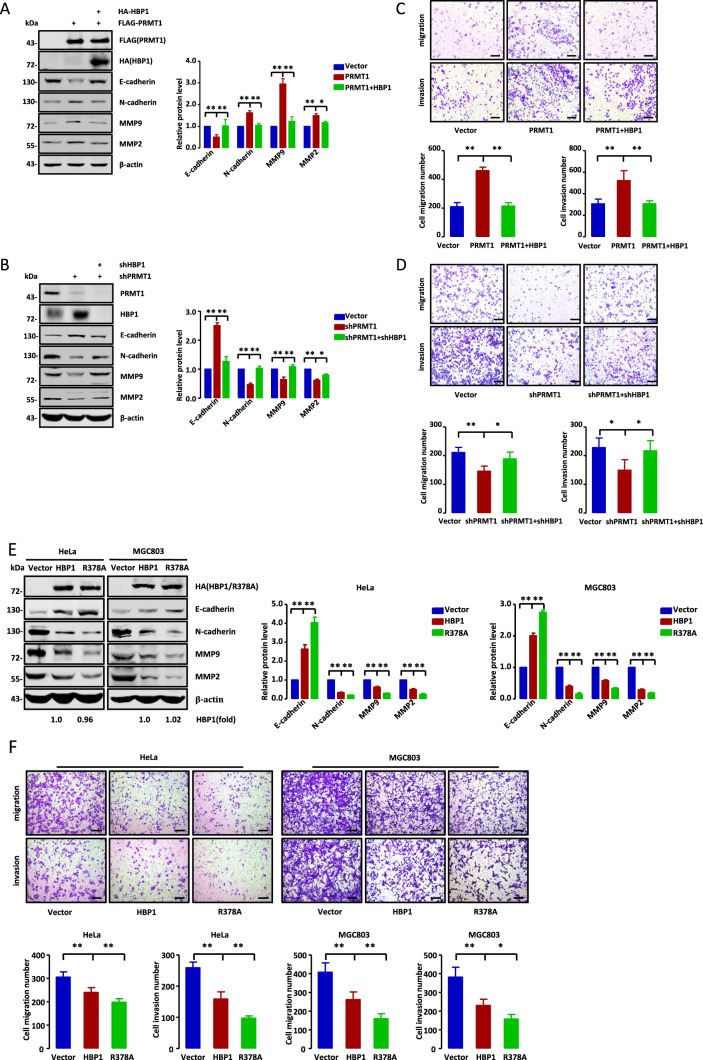

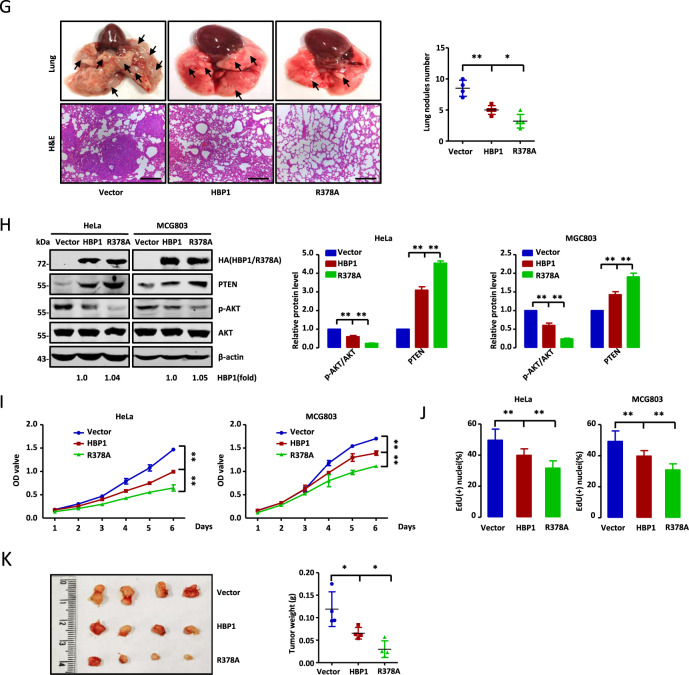
HBP1 methylation alleviates HBP1-mediated suppression of metastasis and growth of tumor cells. **A**–**D** PRMT1-mediated HBP1 methylation participates in PRMT1-induced metastasis in tumor cells. **A** The protein levels of metastasis-related markers were detected by western blotting in HeLa cells transfected with PRMT1 with or without HBP1. **B** The protein levels of metastasis-related markers were detected by western blotting in HeLa cells transfected with shPRMT1 with or without shHBP1. **C** The migratory and invasive potential of HeLa cells transfected with PRMT1 with or without HBP1 were measured by Transwell assay (*n* = 4, one-way ANOVA). Scale bar, 200 µm. **D** The migratory and invasive potential of HeLa cells transfected with shPRMT1 with or without shHBP1 were measured by Transwell assay (*n* = 4, one-way ANOVA). Scale bar, 200 µm. **E**, **G** HBP1 methylation alleviates HBP1-mediated suppression of metastasis of tumor cells. **E** HeLa and MGC803 cells were transfected with empty vector, HBP1 or R378A and the protein levels of metastasis-related markers were verified by western blotting. **F** The migratory and invasive potential of HeLa and MGC803 cells transfected with empty vector, HBP1, or R378A were measured by Transwell assay (*n* = 4, one-way ANOVA). Scale bar, 200 µm. **G** The empty vector, HBP1 and R378A stably transfected HeLa cells were injected into nude mice via the tail vein. Visible lung tumors were quantitatively analyzed (top and right panels). Representative images and hematoxylin and eosin (H&E) staining of metastatic lung tumors are shown (bottom panels) (*n* = 5, one-way ANOVA). Scale bar, 200 µm. **H**–**K** HBP1 methylation alleviates its inhibition of tumor growth. **H** HeLa and MGC803 cells were stably transfected with empty vector, HBP1 or R378A and the levels of PTEN, p-AKT and AKT were measured by western blotting. **I**, **J** Proliferation rates of the cells above were measured by MTT (*n* = 3, one-way ANOVA) (**I**) and EdU (**J**) (*n* = 7, one-way ANOVA) assays. **K** HeLa cells stably transfected with empty vector, HBP1 or R378A were subcutaneously injected into nude mice. Tumor volume and weight were quantitatively analyzed after 4 weeks (*n* = 4, one-way ANOVA). Date were the mean ± SD. *p < 0.05, **p < 0.01.

Bikkavilli et al. reported that PRMT1 can promote metastasis in non-small cell lung cancer by methylating transcription factor Twist1 [22]. Our data identified HBP1 protein as a substrate of PRMT1 that can be methylated at arginine 378 to decrease its stability. Thus, we hypothesized that PRMT1-mediated HBP1 methylation may participate in PRMT1-induced metastasis in tumor cells. As shown in Fig. 3A, overexpression of PRMT1 in HeLa cells induced a dramatic decrease in E-cadherin and a remarkable increase in N-cadherin, MMP9, and MMP2 protein levels. Co-expression of HBP1 could rescue the PRMT1-induced changes in these metastasis marker protein levels, while R378A co-expression reversed the effects of PRMT1 more strongly than wild-type HBP1 co-expression (Fig. S2A, B). The binding ability of PRMT1 and HBP1 was consistent with that of PRMT1 and R378A (Fig. S2C), indicating that methylation did not affect the binding of HBP1 and PRMT1. Moreover, PRMT1 knockdown by shRNA in HeLa cells had the opposite effect on the protein levels of these markers, but had no effect when HBP1 was simultaneously knocked down (Fig. 3B). We next performed Transwell assays with HeLa cells. The results show that PRMT1 overexpression enhanced both the migratory and invasive potential of these cells, whereas co-expression of HBP1 rescued the effects of PRMT1 overexpression (Fig. 3C). Furthermore, the migratory and invasive potential of the cells was inhibited by PRMT1 silencing, while HBP1 silencing rescued this inhibition (Fig. 3D). Collectively, our data suggest that PRMT1-mediated HBP1 methylation facilitates PRMT1-induced metastasis in tumor cells. We speculated that wild-type HBP1 could sequester PRMT1 by physical binding, and partially bound HBP1 could be degraded by PRMT1 methylation, while undegraded HBP1 could partially reverse effects of PRMT1 overexpression. R378A could reverse the effects of PRMT1 overexpression more strongly because it could not be methylated by PRMT1.

To further investigate the role of HBP1 methylation in metastasis, we used a lentiviral expression vector to overexpress wild-type HBP1 or R378A mutant in HeLa and MGC803 cells. HBP1 overexpression resulted in increased E-cadherin and decreased N-cadherin, MMP9, and MMP2 protein levels, and thus inhibited the migration and invasion of the cells. However, overexpression of R378A, which lost its ability to be methylated by PRMT1, resulted in more significantly changed metastasis-related protein levels and inhibited tumor cell migration and invasion rates (Fig. 3E, F). We next investigated the impact of HBP1 methylation on metastasis in vivo. HeLa cells stably expressing empty vector, HBP1, or R378A were injected into 6-week-old female Balb/c nude mice via the tail vein. Two months later, the nude mice were sacrificed and the number of formed lung tumors were examined as a way to analyze the distant metastasis of the HeLa cells. As shown in Fig. 3G, there were significantly fewer lung tumors in the mice injected with R378A-expressing cells compared with the HBP1 and empty vector groups. Furthermore, the xenograft tumors from the R378A-expressing cells resulted in less damage to the lung. These results indicate that the methylation of HBP1 by PRMT1 can alleviate HBP1-mediated suppression of metastasis in tumor cells.

Next, we assessed the effect of HBP1 methylation on its growth-suppressive function. We first detected the expression of PTEN/PI3K/AKT pathway-related proteins in HeLa and MGC803 cells transfected with wild-type HBP1 or R378A. As shown in Fig. 3H, HBP1 transfection increased PTEN and decreased p-AKT protein levels, while R378A transfection resulted in more robust changes to the protein levels. Consistent with this protein expression pattern, HBP1 and R378A could both inhibit cell growth, but R378A had more significant effects as assessed by MTT (Fig. 3I) and EdU incorporation (Fig. 3J) assays. To examine the role of HBP1 methylation in tumorigenesis in animals, 4-week-old BALB/c nude mice were subcutaneously injected with HeLa cells stably expressing empty vector, HBP1, or R378A. R378A expression resulted in a noticeably stronger suppression of tumor growth compared with wild-type HBP1 or empty vector in the xenograft models (Fig. 3K). Overall, our results suggest that HBP1 methylation alleviates its suppression of tumor cell metastasis and growth.

### Pharmacological or enzymatic inhibition of HBP1 methylation suppresses metastasis and growth of tumor cells

**Fig. 4:**
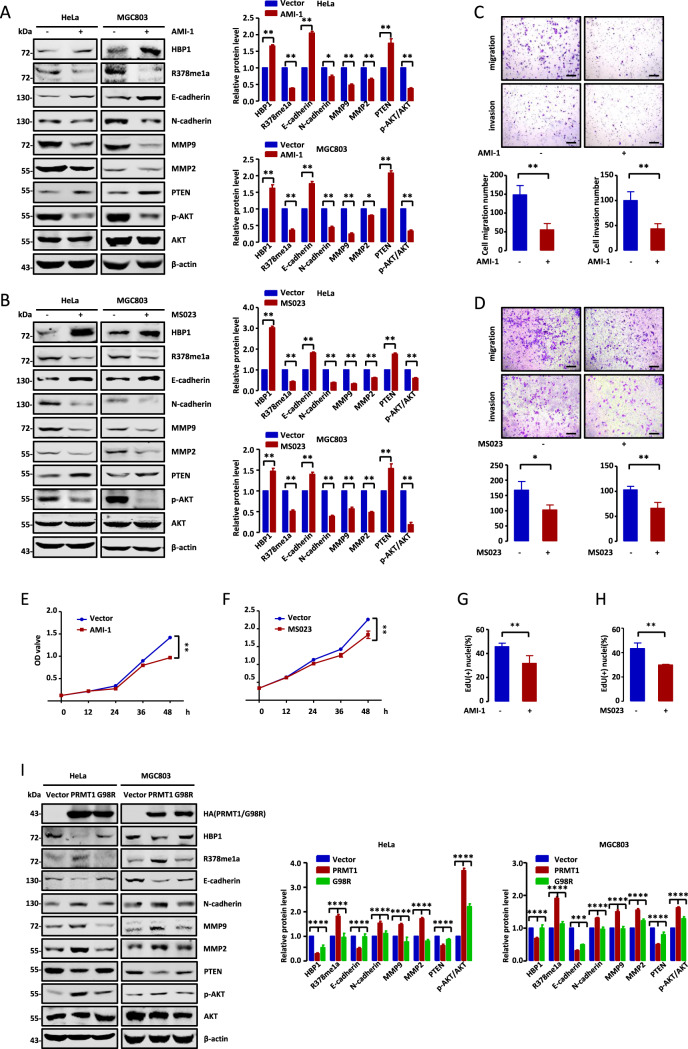

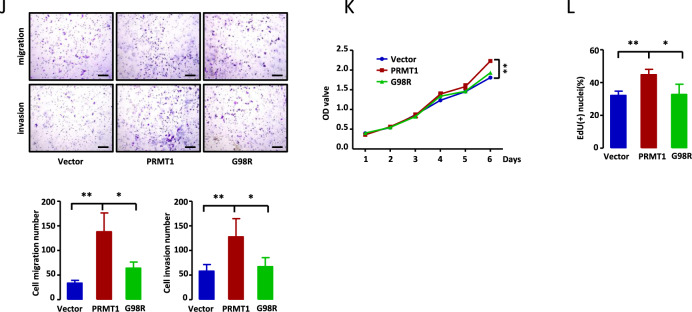
Pharmacological or enzymatic inhibition of HBP1 methylation suppresses tumor cell metastasis and growth. **A**–**H** AMI-1 (Selleckchem, Cat#S7884) and MS023 (MedChemExpress, Cat#HY-19615) suppresses tumor cells metastasis and growth. **A**, **B** The protein levels of metastasis-related and proliferation-related markers were measured by western blotting in HeLa and MGC803 cells treated with AMI-1 (10 µM, 24 h) and MS023 (10 µM, 24 h). **C**, **D** Transwell assays were performed to test the migratory and invasive potential of the cells above (*n* = 5, Student’s *t*-test). Scale bar, 200 µm. **E**, **F** MTT (*n* = 3) and **G**, **H** EdU incorporation assays were performed to test the ability of cell growth in the cells above (*n* = 4, Student’s *t*-test). **I**–**L** G98R suppresses tumor cells metastasis and growth. **I** The protein levels of metastasis-related and proliferation-related markers were measured by western blotting in HeLa and MGC803 cells transfected with empty vector, PRMT1 or G98R. **J** Transwell assays were performed to test the migratory and invasive potential of the cells above (*n* = 4, one-way ANOVA). Scale bar, 200 µm. **K** MTT (*n* = 3, one-way ANOVA) and **L** EdU incorporation assays were performed to test the ability of cell growth in the cells above (*n* = 4, one-way ANOVA). Date were the mean ± SD. *p < 0.05, **p < 0.01.

Because PRMT1-mediated HBP1 methylation can promote metastasis and growth of tumor cells, we next asked if pharmacological inhibition of HBP1 methylation could suppress tumor cell metastasis and growth. We used AMI-1 and MS023, both of specific PRMT1 inhibitors that can block its enzymatic activity [17, 29], to test their effects on cell viability in HeLa and MGC803 cells. As shown in Fig. 4A, B, AMI-1 or MS023 treatment remarkably stabilized HBP1 protein and reduced HBP1 R378 methylation levels. Moreover, E-cadherin and PTEN protein levels were significantly increased, whereas N-cadherin, MMP9, MMP2, and p-AKT protein levels were decreased following AMI-1 or MS023 treatment. According to Transwell, MTT, and EdU incorporation assays, we observed that AMI-1 or MS023 treatment could reduce tumor cell metastasis and growth (Fig. 4C–H).

Subsequently, we utilized PRMT1 mutant G98R, which has lost its enzymatic activity, to test its role in HBP1 methylation and tumor cell metastasis and growth. As shown in Fig. 4I, overexpression of PRMT1, not G98R, induced a dramatic decrease in HBP1 protein levels and increase in HBP1 R378 methylation levels. In addition, we found that N-cadherin, MMP9, MMP2, and p-AKT protein levels were increased, while E-cadherin and PTEN levels were decreased, in PRMT1 overexpression cells but not G98R or empty vector cells. Accordingly, G98R overexpression inhibited tumor cell metastasis and growth compared with PRMT1 overexpression (Fig. 4J–L). Thus, we conclude that pharmacological or enzymatic inhibition of HBP1 methylation can suppress tumor cell metastasis and growth.

### HBP1 methylation alleviates its transcriptional activation of *GSN*

**Fig. 5:**
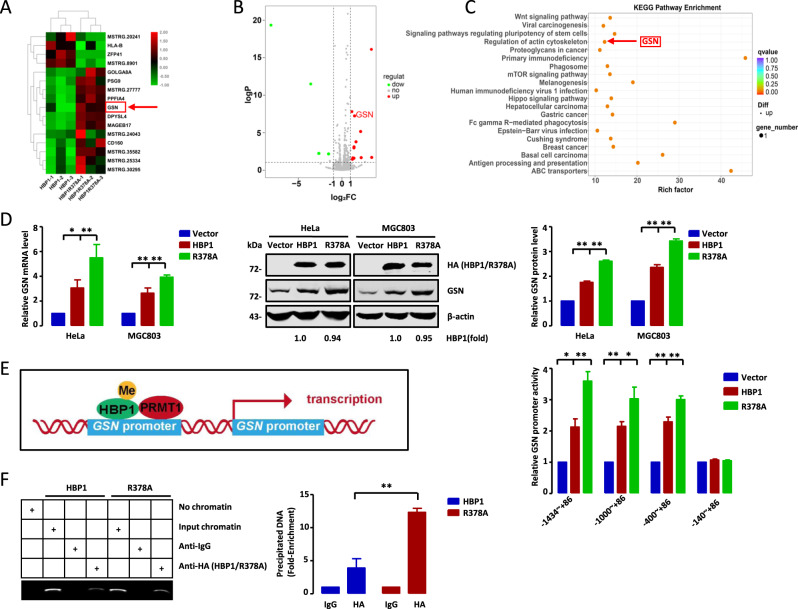
HBP1 methylation alleviates its transcriptional activation of *GSN*. **A**–**C** RNA-seq analysis found that 16 genes were differentially expressed in HeLa cells transfected with HBP1 or R378A. Heatmap (**A**) and volcano plot **B** showed 16 differentially expressed genes between HBP1 and R378A expressing cells with RNA-seq analysis. KEGG enrichment analysis revealed 16 genes enriched in different biological pathways **C**. **D** Methylation of HBP1 at R378 alleviates HBP1 promotion of GSN mRNA and protein levels. GSN mRNA and protein levels were measured with real-time PCR and western blotting in HeLa and MGC803 cells transfected with empty vector, HBP1 or R378A. **E**, **F** Methylation of HBP1 at R378 alleviates its transcriptional activation on *GSN*. **E** HEK293T cells were co-transfected HBP1 or R378A with different *GSN* promoter segment. The luciferase activities were expressed (right panels). **F** ChIP assay was used to test HBP1 direct binding to *GSN* promoter. HEK293T cells were transfected with HBP1 or R378A. Specific binding of HBP1 or R378A to *GSN* promoter (−400 to −140) were measured by specific PCR (left panel) and real-time PCR (right panel). IgG was used as a negative control. Date were the mean ± SD (*n* = 3, one-way ANOVA). *p < 0.05, **p < 0.01.

To investigate the mechanism controlling how HBP1 methylation regulates tumor cell metastasis and growth, we performed RNA sequencing (RNA-seq) analysis in HeLa cells expressing wild-type HBP1 or R378A, then clustered the same or similar gene expression patterns with hierarchical cluster analysis. The heatmap and volcano plot show that 16 differentially expressed genes, including 12 upregulated and 4 downregulated genes, were separated into R378A-expressing cells compared with HBP1-expressing cells (Fig. 5A, B). KEGG pathway enrichment analysis revealed 16 genes enriched in different pathways (Fig. 5C). *GSN*, one of 12 upregulated genes in R378A-expressing cells, immediately became of interest. As an important actin-binding protein, GSN is involved in many biological functions including the regulation of cell structure, motility, growth, and apoptosis, as well as signal transduction and phagocytosis processes [23]. The basic function of GSN is to maintain the balance of G-actin and F-actin levels by severing and capping F-actin filaments. Abnormal increases in F-actin promote reorganization of the actin cytoskeleton, which may ultimately lead to a malignant phenotype. To identify the potential relevance of HBP1 methylation and GSN, we first detected GSN mRNA levels in HeLa and MGC803 cells expressing empty vector, HBP1, or R378A by real-time PCR. As shown in Fig. 5D, R378A-expressing cells showed the highest GSN mRNA levels, while there were moderate GSN mRNA levels in HBP1-expressing cells compared with control cells (left panels). GSN protein levels were consistent with the observed mRNA levels (right panels). These data suggest that the R378A mutant cannot be methylated by PRMT1 and promotes GSN expression, while wild-type HBP1 can be methylated by endogenous PRMT1 and reduces GSN expression at the transcriptional level.

We next questioned, as a transcription factor, if HBP1 could regulate GSN expression by binding to the *GSN* gene promoter. To address this, we co-transfected HEK293T cells with wild-type HBP1 or R378A with *GSN* promoter constructs of distinct length: −1434 to +86, −1000 to +86, −400 to +86, or −140 to +86 bp from the transcriptional start site. As shown in Fig. 5E, both R378A and wild-type HBP1 activated *GSN* promoters −1434 to +86, −1000 to +86, and −400 to +86, but had no effect on the −140 to +86 *GSN* promoter, indicating that the affinity site of R378A and HBP1 is between −400 to −140 bp from the *GSN* transcriptional start site. Additionally, R378A showed stronger transcriptional activation of the *GSN* promoter compared with HBP1, indicating that HBP1 methylation at R378 alleviates its transcriptional activation of *GSN*. To further identify that *GSN* is a direct target of HBP1, we performed chromatin immunoprecipitation (ChIP) assays. The results show that HBP1 can bind directly to the *GSN* promoter and R378A can enhance the binding to the promoter (Fig. 5F). According to the previously reported HBP1 affinity site [30], we constructed a reporter gene with possible HBP1 binding site deletion (Luc-ΔGSN, deletion −296 to −271 bp). As shown in Fig. S3A, HBP1 and R378A mutant activated Luc-GSN which had possible HBP1 binding site, while had no effect on Luc-ΔGSN, indicating that HBP1 binding site is located in the −296 to −271bp region of the *GSN* promoter. To test if co-expression of PRMT1 affected the effect of HBP1 on *GSN* promoter, we co-transfected HBP1, R378A, PRMT1 + HBP1 or PRMT1 + R378A with *GSN* promoter segment into HEK293T cells. As shown in Fig. S3B, the co-transfection of PRMT1 reduced the activation of HBP1 on *GSN* promoter, but did not affect the activation of R378A. Accordingly, the co-transfection of PRMT1 decreased the binding of HBP1 to *GSN* promoter, but did not affect the binding of R378A to *GSN* promoter (Fig. S3C). In addition, using western blotting and real-time PCR, we found that PRMT1 overexpression decreased GSN protein and mRNA levels (Fig. S3D), indicating PRMT1 itself can inhibit *GSN* gene expression. Our data clearly demonstrate that methylation of HBP1 at R378 can alleviate its transcriptional activation of *GSN* by reducing HBP1 binding to the *GSN* promoter, thus decreasing GSN expression levels.

### Methylation of HBP1 promotes actin cytoskeleton remodeling by downregulating GSN

**Fig. 6:**
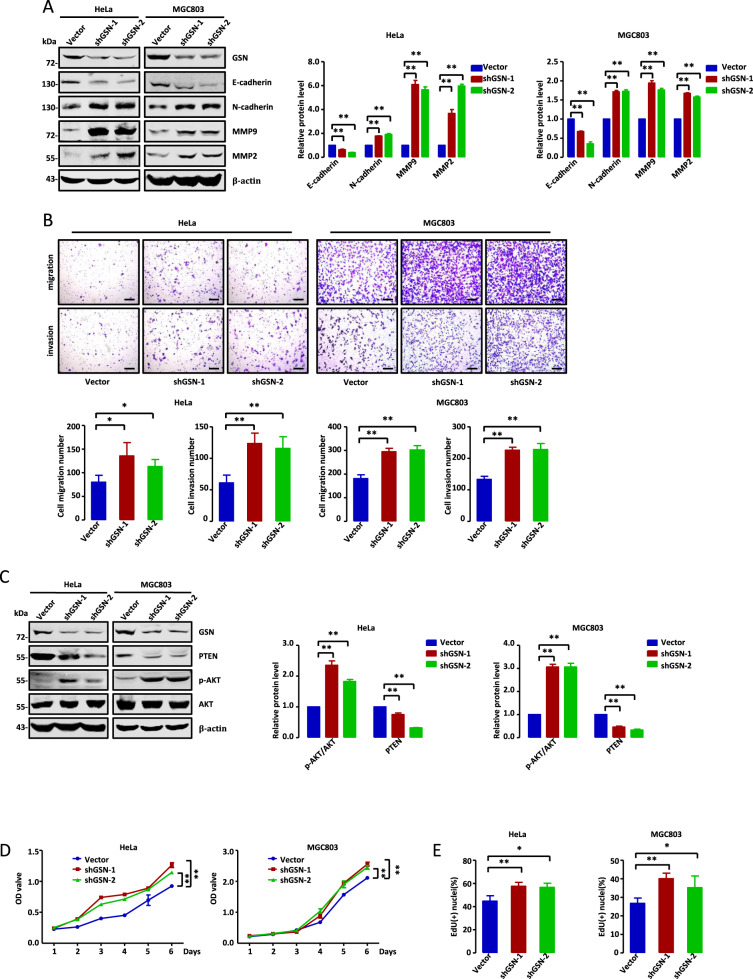

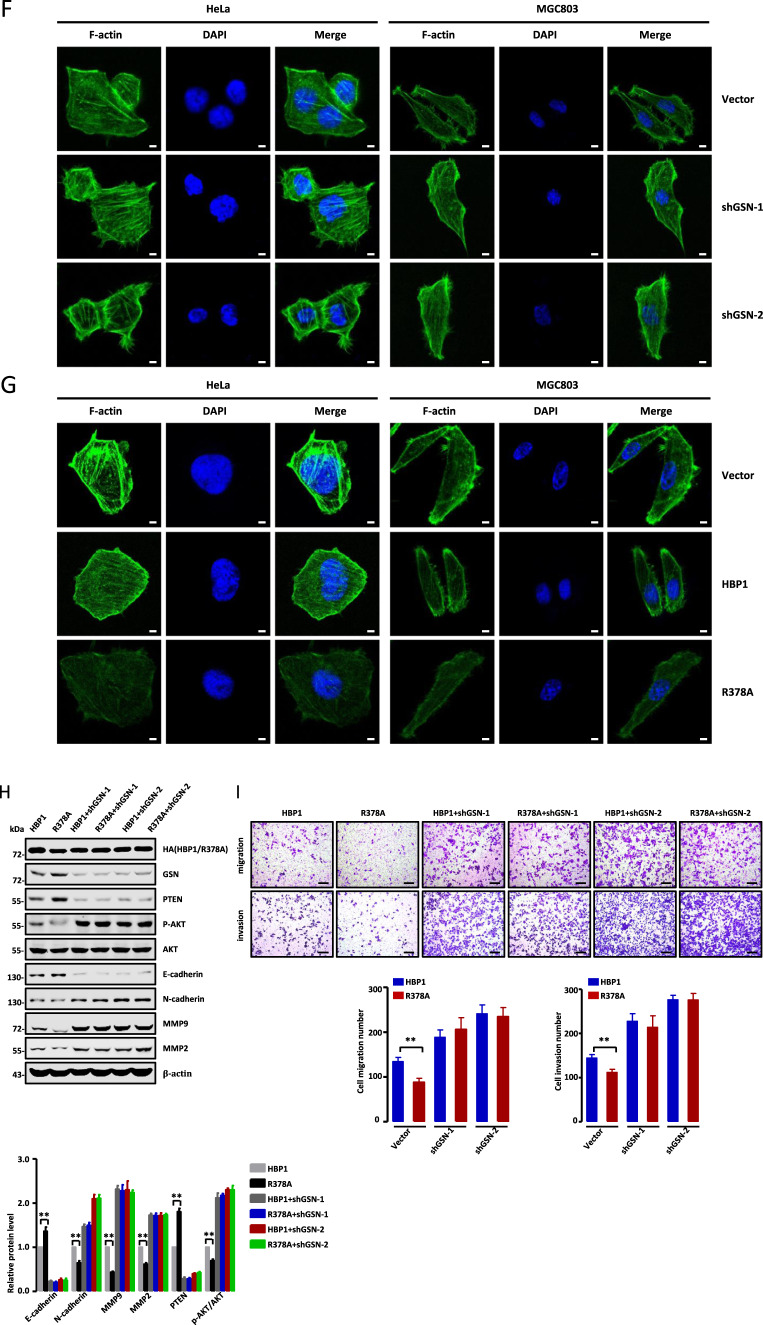

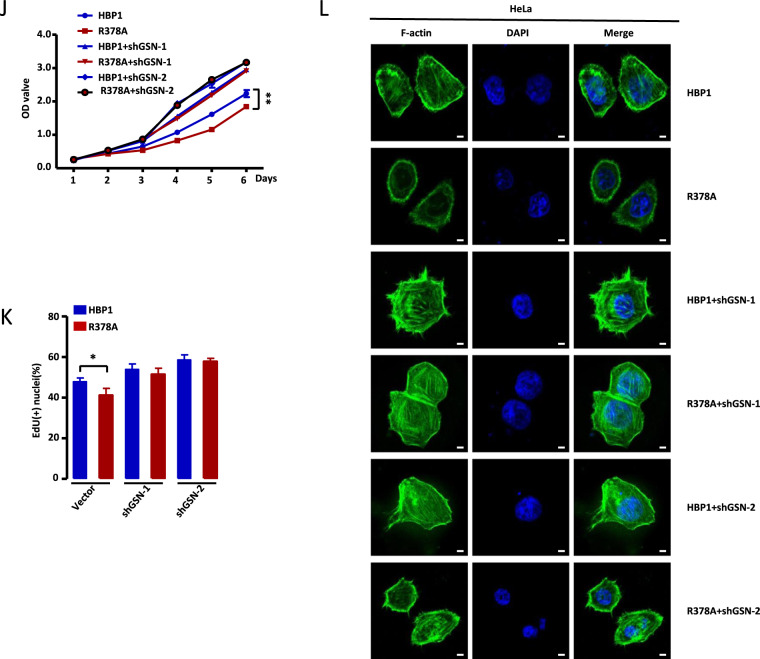
Methylation of HBP1 promotes actin cytoskeleton remodeling by downregulating GSN. **A**, **C** GSN knockdown inhibits cell migration and invasion of HeLa and MGC803 cells. **A** The protein levels of metastasis-related markers were measured by western blotting in HeLa and MGC803 cells transfected with empty vector, GSNshRNA1 or GSNshRNA2. **C** Transwell assays were performed to test the migratory and invasive potential of the cells above (*n* = 4, one-way ANOVA) (**A**). Scale bar, 200 µm. **B**, **D**, **E** GSN knockdown inhibits cell proliferation in HeLa and MGC803 cells. **B** The levels of proliferation-related proteins were measured by western blotting in the cells above (**A**). **D** MTT (*n* = 3, one-way ANOVA) and (**E**) EdU incorporation assays were performed to test the ability of cell growth in the cells above (*n* = 4, one-way ANOVA) (**A**). **F** GSN knockdown promotes filaments formation. F-actin were stained with phalloidin in the cells above (**A**). **G** Methylation of HBP1 alleviates its inhibition of filaments formation. HeLa and MGC803 cells were stably overexpressing empty vector, HBP1 or R378A and the levels of F-actin were analyzed by immunofluorescence intensity. Scale bar, 5 µm. **H**–**K** Methylation of HBP1 alleviates its inhibition of metastasis and growth of HeLa cells through down-regulating GSN expression. **H** HeLa cells stably expressing HBP1, R378A, HBP1 + shGSN-1, R378A + shGSN-1, HBP1 + shGSN-2, and R378A + shGSN-2 individually, the protein levels of metastasis-related and proliferation-related markers were measured by western blotting. **I** Transwell assays were performed to test the migratory and invasive potential of the cells above (*n* = 4, one-way ANOVA) (**H**). Scale bar, 200 µm. **J** MTT (*n* = 3, one-way ANOVA) and (**K**) EdU incorporation assays were performed to test the ability of cell growth in the cells above (*n* = 4, one-way ANOVA) (**H**). **L** Methylation of HBP1 promotes filaments formation by downregulating GSN. F-actin were analyzed by immunofluorescence intensity with the cells above (**H**). Scale bar, 5 µm. Date were the mean ± SD. *p < 0.05, **p < 0.01.

Khurana et al. previously reported that GSN knockout increased the levels of actin filaments, as analyzed by fluorescence intensity of F-actin. This eventually led to mitochondrial defects and necroptosis in intestinal epithelial cells [31]. To further determine the role of GSN in the metastasis and growth of tumor cells, we established two stably transfected cell lines with lower GSN expression using lentiviral vectors expressing a GSN-targeting shRNA, designated as shGSN-1 and shGSN-2. As shown in Fig. 6A, B, GSN knockdown by shGSN-1 or shGSN-2 in HeLa and MGC803 cells affected the expression levels of metastasis-related and PTEN/PI3K/AKT pathway-associated proteins. Transwell assays suggested that GSN knockdown significantly increased cell migration and invasion (Fig. 6C). The MTT and EdU assays indicated that GSN knockdown could promote cell growth (Fig. 6D, E). To assess the role of GSN in cytoskeleton remodeling, HeLa and MGC803 cells transfected with empty vector, shGSN-1, or shGSN-2 were stained with phalloidin and observed by confocal microscopy. The results revealed that GSN knockdown led to an obvious increase of F-actin staining (Fig. 6F), indicating that GSN knockdown promoted filament formation. Overall, our data suggest that GSN acts as a growth inhibitor in these cell lines, and is involved in actin cytoskeleton remodeling in tumor cells.

Next, we assessed the effects of HBP1 methylation on the actin cytoskeleton. HeLa and MGC803 cells were transfected with empty vector, wild-type HBP1, or R378A separately and stained with phalloidin. As judged by F-actin staining, R378A-transfected cells had the lowest fluorescence intensities, while wild-type HBP1 transfection resulted in lower fluorescence intensities compared with the control vector (Fig. 6G). These findings indicate that R378A significantly inhibited filament formation, while HBP1 that could be methylated at R378 by endogenous PRMT1 alleviated R378A-mediated inhibition of filament formation. Our results suggest that HBP1 methylation may accelerate rearrangement of the cytoskeleton by promoting filament formation, and ultimately support the growth and metastasis of tumor cells.

To further examine the role of GSN in HBP1 methylation-induced metastasis and cell proliferation, we constructed HeLa cells stably expressing wild-type HBP1, R378A, HBP1 + shGSN-1, R378A + shGSN-1, HBP1 + shGSN-2, or R378A + shGSN-2. As shown in Fig. 6H, stable expression of R378A increased E-cadherin protein levels and decreased N-cadherin, MMP9, and MMP2 protein levels compared with stable expression of wild-type HBP1. However, GSN knockdown with shRNAs completely reversed the observed changes in metastasis-related markers. Similar results were obtained from Transwell experiments, which revealed that the cells stably overexpressing R378A have lower migration and invasion rates compared with cells overexpressing wild-type HBP1. Again, this inhibition was abolished by GSN knockdown (Fig. 6I). These data indicate that R378A inhibited migration and invasion of HeLa cells by upregulating GSN expression, whereas HBP1 methylation alleviated these effects. Furthermore, we found that PTEN and p-AKT protein levels were remarkably increased and decreased, respectively, in R378A overexpression cells compared with wild-type HBP1 overexpression cells. GSN knockdown disturbed this protective effect (Fig. 6H). MTT and EdU assays further demonstrated that R378A-induced growth inhibition was completely reversed by GSN knockdown (Fig. 6J, K). Similar results were observed with F-actin staining, in which R378A overexpression led to a more effective repression of F-actin fluorescence intensity than HBP1 overexpression. This observed repression was rescued by GSN knockdown (Fig. 6L). Hence, our results suggest that methylation of HBP1 can promote actin cytoskeleton remodeling, thus inducing growth and metastasis of tumor cells by downregulating GSN expression.

### Methylated HBP1-mediated actin cytoskeletal remodeling promotes glycolysis in tumor cells

Usually, the motile aggressive tumor cells exhibit increase glycolysis to obtain more energy. Recent studies have unraveled that mechanical force-induced actin cytoskeleton remodeling promotes metabolic activity, especially glycolysis in tumor cells [32–34]. So we ask question whether methylated HBP1-mediated actin cytoskeletal remodeling promotes glycolysis in tumor cells? Indeed, GSN knockdown showed an obvious increase of glucose uptake, lactate production, and cell medium acidification in HeLa cells (Fig. S4A), indicating that GSN knockdown increases glycolytic rate in cancer cells. Meanwhile, R378A-transfected cells had the lowest level of glycolysis, while wild-type HBP1 transfection resulted in lower level of glycolysis compared with the control vector (Fig. S4B). Interestingly, this protective effect was disturbed by GSN knockdown (Fig. S4C). Taken together, these results suggest that methylation of HBP1 can promote glycolysis by accelerate rearrangement of the cytoskeleton, thereby provide more energy for growth and metastasis of tumor cells.

### Elevated expression of methylated HBP1 and reduced levels of GSN in cervical cancer samples are associated with poorer clinical outcomes

**Fig. 7:**
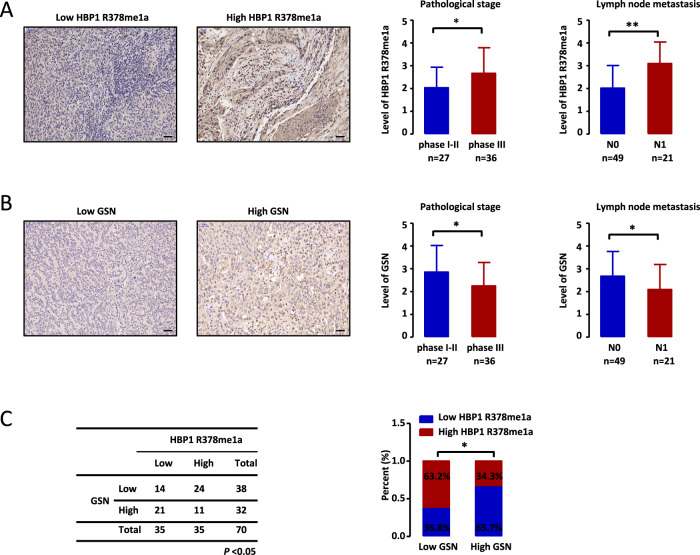
Elevated expression of methylated HBP1 and reduced levels of GSN in cervical cancer samples are associated with poorer clinical outcomes. **A**–**C** High level of HBP1 methylation and low GSN expression relate to poor prognosis. **A**, **B** IHC assays were performed using anti-HBP1 R378me1a and anti-GSN in 70 cervical cancer tissue samples. Representative images were presented. Scale bar, 50 µm. (*n* = 70, Student’s *t*-test) **C** Correlation between HBP1 R378me1a and GSN expression was examined by Pearson correlation coefficient test.

To define whether HBP1 methylation can contribute to cancer progression, we used the HBP1 R378me1a antibody to detect the levels of methylated HBP1 in 70 cervical cancer tissue samples. Immunohistochemistry (IHC) staining results indicated that higher levels of methylated HBP1 were significantly related to higher pathological stage and positive lymph node metastasis (Fig. 7A). Given that GSN knockdown promoted cell growth and metastasis in HeLa and MGC803 cells, GSN protein levels were also detected by IHC staining. The staining intensity analysis revealed a positive correlation between lower GSN expression levels and poorer prognosis (Fig. 7B). These results corroborate data from The Cancer Genome Atlas (TCGA) database, which also show that lower levels of GSN correlate with worse overall survival (Fig. S5). Importantly, we also found that the levels of methylated HBP1 negatively correlated with GSN expression (Fig. 7C). Taken together, our results demonstrate that elevated expression of methylated HBP1 and reduced levels of GSN in cervical cancer are associated with poorer prognosis.

## Discussion

**Fig. 8:**
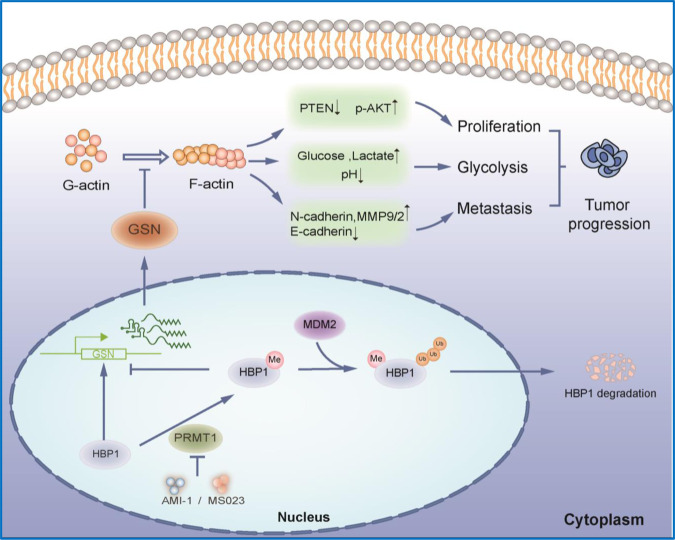
Model of methylation HBP1 by PRMT1 regulating GSN-mediated actin cytoskeleton remodeling in tumor progress. HBP1 can be methylated by PRMT1 at R378. The methylation of HBP1 decreases the stability of HBP1 protein via accelerating its ubiquitination and proteasomal degradation, thereby reduces its transcriptional activation on *GSN*, downregulates GSN expression and leads to F-actin abnormally increasing. The excessive accumulation of F-actin promotes remodeling of the actin cytoskeleton and affects the metastasis and proliferation-related protein levels, and it will bring enormous impact on cancer cells malignant phenotype and promote tumor progression.

The previous studies reported that HBP1 is regulated by multiple types of PTMs, including phosphorylation, acetylation, and ubiquitination. These PTMs of HBP1 have important roles in senescence and tumorigenesis [8, 12, 13]. In this study, we discovered a novel PTM of HBP1 and validated that HBP1 can be methylated at R378 by PRMT1. This methylation diminishes the stability of the HBP1 protein by accelerating its ubiquitination and proteasomal degradation. Methylation of HBP1 at R378 can alleviate its repressive effects on metastasis and tumorigenesis by downregulating GSN expression. Mechanistically, methylation of HBP1 weakens its ability to bind to the *GSN* promoter. This alleviates its transcriptional activation of *GSN*, thereby downregulating GSN expression and leading to an abnormal increase in F-actin levels. This excessive accumulation of F-actin in turn promotes remodeling of the actin cytoskeleton and affects levels of growth, metastasis-related proteins and glycolysis-related indicators. These molecular events therefore impact cancer cell malignant phenotypes and promote tumor progression. In addition, we also demonstrated that high amounts of methylated HBP1 and low GSN expression levels positively correlate with poor prognosis of cervical cancer patients, indicating that methylated HBP1-GSN axis is critical for tumorigenesis, and targeting this axis may provide a new therapeutic strategy for treating cancers. Our results are summarized in a schematic model depicted in Fig. 8.

PRMT1 reportedly contributes to as much as 85% of all cellular PRMT activity [35]. It is expressed in all embryonic and adult tissues, demonstrating the widespread importance of this enzyme in cellular functions [15, 36–38]. PRMT1 has a broad spectrum of non-histone substrates that are involved in crucial cellular processes such as transcription, cell metabolism, and DNA repair [14–16]. PRMT1-mediated methylation of arginine residues in transcription factors has been linked to cancer progression [16]. PRMT1 may act as either an activator or repressor, depending on the specific transcription factor and methylation site. Yamagata et al. demonstrated that PRMT1 can methylate FOXO1 at R248 and R250. This blocks AKT-mediated phosphorylation of FOXO1, thus inhibiting FOXO1 protein degradation and promoting its transcriptional activity [19]. However, PRMT1 can repress the transcriptional activity of C/EBPα by methylating it at R35, R156, and R165, which leads to rapid growth of breast cancer cells [20]. Therefore, PRMT1 is able to fine-tune the expression of various genes by methylating certain transcription factors. In this study, we identified HBP1 as a novel substrate of PRMT1. PRMT1-mediated methylation of HBP1 alleviates its protein stability and transcriptional activation of *GSN*. This thereby promotes actin cytoskeleton remodeling and causes HBP1 to lose its effects on metastasis and tumor inhibition. Interestingly, a previous study reported that multiple substrates of PRMT1 contain an RGG/RG motif for PRMT1 targeting [39], which is not present in the HBP1 protein. In fact, recent reports found that many non-histone proteins have methylation sites not located in the RGG/RG motif, such as EZH2, HSP70, and FLT3 [40–42], indicating that those proteins without the canonical PRMT1 methylation motif may be targeted and methylated by PRMT1.

As a tumor suppressor, HBP1 expression levels are often low in various cancers [30, 43–47]. In this study, we found that HBP1 can be methylated at R378 by PRMT1. Methylated HBP1 protein is less stable compared with non-methylated HBP1, which may explain why HBP1 protein levels are low in diverse cancer types. We therefore hypothesized that inhibiting HBP1 methylation could be used as a novel cancer treatment method. Our experiments revealed that two PRMT1-specific inhibitors (AMI-1 and MS023) could decrease HBP1 methylation and increase HBP1 protein levels, thereby enhancing HBP1-mediated suppression of tumor growth and metastasis. These findings suggest that inhibiting the PRMT1-HBP1 axis may have therapeutic value in cancer. Additionally, high amounts of HBP1 methylation is associated with poor prognosis of cancer patients. Our study also verified that HBP1 methylation can influence expression of its target genes, such as *p53*, *p21*, *p16*, and *DNMT1* (Fig. S6), which may play a vital role in cell metabolism. With this diverse set of HBP1 interactions and targets in cells, it will be of great importance to examine the role of HBP1 methylation in tumor initiation and progression.

Maintaining actin cytoskeleton integrity is essential for preventing cell transformation, and several reports have provided evidence that GSN mediates actin cytoskeleton remodeling by regulating actin filament formation [23]. However, the mechanism of transcriptional regulation of GSN remained elusive. In this study, we identified GSN as a novel target of HBP1. HBP1 methylation downregulates GSN expression levels. The methylated HBP-GSN axis could contribute to actin cytoskeleton remodeling and further promote tumorigenesis. As an actin-binding protein, GSN participates in diverse biological processes by regulating the actin skeleton. The previous study reported that the GSN protein levels are lower in some human cancer cells, including breast, bladder, gastric, lung, and oral cancers, relative to healthy controls [23, 27, 48, 49]. These observations suggest that GSN acts as a growth inhibitor. Our results support this, as GSN knockdown promoted the metastasis, growth and glycolysis of cancer cells and low levels of GSN expression were associated with poor prognosis in cervical cancer patients. In fact, there is growing evidence that the function of GSN is multidimensional in various cancers [23, 25, 48, 50, 51]. GSN acting as a growth inhibitor or oncoprotein depends on the specific pathological conditions and carcinoma type. The mechanism controlling this apparent dual function for GSN in cancers needs to be explored further in the future.

In conclusion, we demonstrate that the methylation of HBP1 by PRMT1 can regulate GSN-mediated actin cytoskeleton remodeling. Importantly, we provide evidence that a novel PRMT1-HBP1-GSN regulatory pathway plays a major role in cancer development and metastasis. Targeting this axis may become a potential new strategy for the treatment of malignant cancers.

## Materials and methods

### Cell culture, transfection, and lentivirus infection

HEK293T, HeLa and MGC803 cells were purchased from ATCC and cultured at 37 °C, 5% CO_2_ in DMEM supplemented with 10% fetal bovine serum (FBS). Cells were transfected using TurboFect transfection reagent (Thermo Scientific) according to the manufacturer’s instructions and harvested 48 h after transfection. The lentivirus plasmid pLL3.7-shHBP1 or pLL3.7-shPRMT1 were transfected to obtain the shHBP1 or shPRMT1 stable cell line. The primers’ sequences used in this study were listed in Supplementary Information (Table 1). To overexpress HBP1 or PRMT1 stably, the entire coding region of the HBP1 or PRMT1 gene was placed into pLVX-IRES-puro lentiviral vector. Cells were selected with puromycin for 1 week and then analyzed for overexpression efficiency.

### Western blotting and antibodies

Cells were lysed in RIPA buffer (Thermo Scientific) including protease inhibitor cocktail (Sigma) and protein concentrations were measured utilizing the BCA protein assay kit (Pierce). A total of 25–50 μg of protein was separated by SDS-PAGE and transferred to nitrocellulose membranes (Pall). The primary antibodies and secondary antibodies which were used are shown in Supplementary Information (Table 2). The antigen of R378me1a antibody was SSMARQRR(me)ASLS.

### Real-time PCR

Total RNA was extracted using the RNAsimple Total RNA kit (Tiangen). Quantitative RT-PCR and real-time PCR were performed utilizing ReverAid FirstStrand cDNA Synthesis kit (Vazyme) and Maxima SYBR Green qPCR Master Mix (Vazyme) according to the manufacturer’s instructions. The primers’ sequences used in this study were listed in Supplementary Information (Table 1).

### Immunoprecipitation and mass spectrometry

Cells were harvested and lysed with IP lysis buffer (25 mM Tris-HCl (pH 7.4), 150 mM NaCl, 1% Nonidet P-40, 1 mM EDTA, and 5% glycerol) including protease inhibitor cocktail (Sigma) at 4 °C for 1 h. The samples were incubated with Protein A-Sepharose (GE Healthcare) and the indicated antibody on a rocking platform overnight at 4 °C. The following day, the IPs were washed with IP lysis buffer for three times and boiled in 2 × SDS loading buffer for 10 mins at 95 °C. For Mass spectrometry, HEK293T cells were co-transfected indicated plasmids and subjected to immunoprecipitation. Elutes were resolved on SDS-PAGE and stained with Coomassie blue staining. The protein band corresponding to HBP1 was excised and subjected to mass spectrometry analysis.

### GST pull-down assay

The GST-tagged proteins or GST alone which purified from Escherichia coli strain BL21 (DE3) were incubated with glutathione-Sepharose beads (GE Healthcare) on a rocking platform overnight at 4 °C. The following day, the samples were incubated with His-tagged proteins for 4 h at 4 °C. Then, the beads were washed three times with GST elution buffer and subjected to western blotting with the indicated antibodies.

### In vitro methylation assay

The purified GST-PRMT1 and His-HBP1 were incubated together with Histone methyltransferase (HMT) buffer (25 mM Tris-HCl pH 8.8, 25 mM NaCl, 2 μM SAM). The reactions were incubated at 37 °C for 2 h.

### In vivo ubiquitination assay

Cells were transfected with plasmids for 36 h and treated with 10 μM MG132 for 6 h before harvesting. The samples were immunoprecipitated and incubated with indicated antibody on a rocking platform overnight at 4 °C. The next day, the samples were washed three times and subjected to western blotting with the indicated antibodies.

### Protein half-life assay

PRMT1 overexpression or shPRMT1-transduced HeLa cells were treated with the cycloheximide (CHX) for 0, 30, 60, and 90 min separately at final concentration of 100 μg/ml. The endogenous HBP1 levels were detected by western blot and normalized to β-actin with using Image J software. The half-life of HBP1 protein t1/2 measurements were performed as described previously [11].

### Immunofluorescence staining

Transfected cells were cultured in 3.5 cm confocal dishes and washed with PBS at 37 °C for three times. Then, cells fixed with 4% paraformaldehyde for 15 min and permeabilized (using 0.2% Triton X-100 in PBS) for 15 min under room temperature. After washing with PBS for three times, cells were blocked for 1 h with 1% bovine serum albumin in PBS under room temperature and incubated with primary antibodies overnight in 4 °C. The following day, cells were incubated with the secondary antibodies conjugated with AlexaFlour488 (anti-rabbit IgG). For F-actin staining, cells were washed, fixed, permeabilized and incubated with phalloidin (cytoskeleton). DNA was stained with DAPI at final concentration of 1 μg/ml. Images were photographed using a ZEISS fluorescence microscope.

### Cell migration and invasion assay

About 5 × 10^4^ cells were used for assessing cell migration and invasion. Cells were cultured with serum-free DMEM medium in the Transwell inserts (Corning) containing 8 μm permeable pores and allowed to migrate into lower chambers which filled with DMEM supplemented with 10% FBS. For the invasion assay, the Transwell inserts were covered with Matrigel. 24 h later, the metastatic cells were fixed with 4% paraformaldehyde and stained by 0.1% crystal violet. Then the cells were photographed by the microscopy (Leica) and counted by image J software.

### MTT and EdU incorporation

About 3 × 10^3^ cells were seeded in 96-well plates for 6 days. 15 μl MTT solution (5 mg/ml) was added to each well and incubated at 37 °C for 4 h. The cell proliferation ability was measured by the intensity of absorbance at 490 nm. The EdU incorporation assay was assessed using EdU kit (Ribobio) and cells were photographed by the microscopy (Leica) at least five randomly chosen fields.

### Reporter gene assay

HEK293T cells were plated on 12-well plates and transfected with plasmids about 30–48 h before harvesting. The luciferase activity was measured by the Dual-Luciferase Reporter Assay kit (Promega). Each assay was performed at least three times.

### Chromatin immunoprecipitation (ChIP)

ChIP assays were performed as described previously [6]. The ChIP primer sequences were listed in Supplementary Information (Table 1).

### Glycolytic activity assay

For glucose uptake assay, cells were treated with 2-NBDG (50 µM, MedChemExpress) for 1 h, the level of glucose uptake was measured by fluorescence intensity (Varioskan™ LUX, Thermo Scientific). For lactate production assay, cells were plated on 6-well plates and cultured for 24 h. The lactate in the culture medium was measured by Lactic Acid Assay Kit (Nanjing Jiancheng Bioengineering Institute, Nanjing, China). The pH of the media was measured by PB-10 (Sartorius).

### Animal studies

To evaluate the role of HBP1 methylation in tumorigenesis, six-week-old female Balb/c nude mice were purchased from Peking University Health Science Animal Center and complied with the Committee on the Ethics of Animal Experiments of Peking University. About 3 × 10^6^ stable transfected cells were suspended in 200 μl PBS and subcutaneously injected into mice. After 3–4 weeks, mice were sacrificed, and the tumors were measured. As for lung metastasis model, about 1 × 10^6^ stable transfected cells were suspended in 200 μl PBS and injected via the tail vein. Two months later, mice were sacrificed, and lung were analyzed for distant metastasis.

### Immunohistochemistry analysis

Cervical cancer samples (Cat No. CXC1502) from patients were purchased from Fanpu Biotech, Inc (Guangxi, China). IHC of methylated HBP1 was performed using homemade R378me1a antibody (1:500) which obtained from willget (Shanghai, China). A total score of protein expression was classified into four groups (1, low staining; 2, weak staining; 3, moderate staining; 4, strong staining) according to the percentage of immunopositive cells and immunostaining intensity. We defined that 3/4 as high expression and 1/2 as low expression.

### Statistical analysis

Statistical analyses were performed using SPSS software. The two-tailed, Student *t* test was used to compare two groups and one way ANOVA was performed to the comparisons among more than two groups. All data were expressed as mean ± SD from at least three-independent experiments. *P* value < 0.05 was considered as statistically significant. *p < 0.05, **p < 0.01.

## Supplementary information

Supplementary Information

Figure S1

Figure S2

Figure S3

Figure S4

Figure S5

Figure S6

## Data Availability

All data generated or analyzed during this study are available from the corresponding author on reasonable request.
