# Supplementary material for: Methylation of HBP1 by PRMT1 promotes tumor progression by regulating actin cytoskeleton remodeling
Source: Oncogenesis. 2022 Aug 8;11(1):45. doi: 10.1038/s41389-022-00421-7 (PMC9360041; doi:10.1038/s41389-022-00421-7)
Supplement: Supplementary file 1 — Supplementary Information [file 41389_2022_421_MOESM1_ESM.docx]

**Supplementary Information**

**Methylation of HBP1 by PRMT1 promotes tumor progression by regulating actin cytoskeleton remodeling**

Jiyin Wang^1^, Ruixiang Yang^1^, Yuning Cheng^1^, Yue Zhou^1^, Tongjia Zhang^1^, Shujie Wang^1^, Hui Li^1^, Wei Jiang^1^, Xiaowei Zhang^1,#^

**Table 1. Information of the oligonucleotides**

| Oligonucleotides | Forward primer(5’-3’) | Reverse primer(5’-3’) |
| --- | --- | --- |
| Human HBP1 real-time PCR | TGAAGGCTGTGATAA  TGAGGAAGAT | CATAGAAAGGGTGGT  CCAGCTTA |
| Human PRMT1 real-time PCR | CCTTCACCTCCCCGTT  CTG | CCAGGGCGTGCACGT  AGT |
| Human GAPDH real-time PCR | CCATGGAGAAGGCTG  GGG | CAAAGTTGTCATGGA  TGACC |
| Human GSN real-time PCR | AGGTGTGGCATCAGG  ATTCA | ATTGCTGTTGGAACC  ACACC |
| GSN ChIP | CGGAATGAGCTGGGA  ATCGCTCTTCC | GAGGCCCGGAGCACGGAGG |
| shHBP1 targeting sequence | ACTGTGAGTGCCACTTC |  |
| shPRMT1 targeting sequence | CCGGCAGTACAAAGACTACA |  |
| shGSN targeting sequence-1 | AACGATGCCTTTGTTCTGAAA |  |
| shGSN targeting sequence-2 | CAGCTACATCATTCTGTACAA |  |
|  |  |  |

**Table 2. Information of the antibodies**

| Antibodies | SOURCE | IDENTIFIER |
| --- | --- | --- |
| HBP1 | Proteintech | Cat#11746-1-AP |
| GSN | Proteintech | Cat #11644-2-AP |
| N-cadherin | Proteintech | Cat# 22018-1-AP |
| MMP2 | Proteintech | Cat#10373-2-AP |
| MMP9 | Proteintech | Cat#10375-2-AP |
| p16 | Proteintech | Cat#10883-1-AP |
| His | Proteintech | Cat#66005-1-Ig |
| Mono-Methyl Arginine | Cell Signaling Technology | Cat#8015 |
| Asymmetric Di-Methyl Arginine | Cell Signaling Technology | Cat#13522 |
| PTEN | Cell Signaling Technology | Cat#9188 |
| p-AKT | Cell Signaling Technology | Cat#4060 |
| AKT | Cell Signaling Technology | Cat#9272 |
| PRMT1 | Cell Signaling Technology | Cat#2449 |
| E-cadherin | Santa Cruz | Cat# sc-8426 |
| DNMT1 | Santa Cruz | Cat# sc-271729 |
| p53 | Santa Cruz | Cat# sc-126 |
| IgG | Santa Cruz | Cat# sc-2025 |
| Muti-Ub | MBL | Cat# D058-3 |
| p21 | MBL | Cat# K0081-3 |
| FLAG | Sigma-Aldrich | Cat# F1804 |
| HA | Covance | Cat#MMS101P |
| GST | M&C Gene Technology | Cat#IT003 M |
| β-actin | ABclonal | Cat#AC038 |
| R378me1a | willget | N/A |
| secondary mouse IgG DyLight 800 | Rockland. | Cat#610-145-121 |
| secondary rabbit IgG DyLight 800 | Rockland. | Cat#611-145-002 |
|  |  |  |

**Supplementary figure legends:**

Figure S1. HEK293T cells were co-transfected FLAG-HBP1 with or without HA-PRMT1 and purified by protein A-Sepharose. The reaction products were resolved by SDS-PAGE and gels were stainned with Coomassie blue. The purified protein was retrieved and analyzed by mass spectrometry.

Figure S2. PRMT1-mediated HBP1 methylation participates in PRMT1-induced metastasis in tumor cells. (A) The protein levels of metastasis-related markers were detected by western blotting in HeLa cells transfected with PRMT1 with or without HBP1/R378A. (B) The migratory and invasive potential of HeLa cells transfected with PRMT1 with or without HBP1/R378A were measured by Transwell assay (n=4, one-way ANOVA). Scale bar, 200 µm. (C) PRMT1 interacts with HBP1/R378A. HEK293T cells were co-transfected with HA-HBP1/R378A and FLAG-PRMT1. The Co-IP assay was carried out by using anti-FLAG antibody and followed by western blotting with anti-HA antibody. *p < 0.05, **p < 0.01.

Figure S3. PRMT1 regulates the expression of GSN. (A-C) Methylation of HBP1 at R378 alleviates its transcriptional activation on *GSN*. (A) HEK293T cells were co-transfected HBP1, R378A, PRMT1+HBP1 or PRMT1+R378A with *GSN* promoter segment (Luc-GSN) or mutant *GSN* promoter segment (Luc-**Δ**GSN). The luciferase activities were expressed. (B) HEK293T cells were co-transfected HBP1, R378A, PRMT1+HBP1 or PRMT1+R378A with *GSN* promoter segment. The luciferase activities were expressed. (C) ChIP assay was used to test HBP1 direct binding to GSN promoter. HEK293T cells were transfected with HBP1, R378A, PRMT1+HBP1 or PRMT1+R378A. Specific binding of HBP1 or R378A to *GSN* promoter (-400 to -140) were measured by specific PCR (left panel) and real-time PCR (right panel). IgG was used as a negative control (n=3, one-way ANOVA). (D) GSN mRNA and protein levels were measured with real-time PCR and western blotting in HeLa and MGC803 cells transfected with empty vector or FLAG-PRMT1 (n=3, Student’s *t*-test). Date were the mean±SD. *p < 0.05, **p < 0.01.

Figure S4. Methylation of HBP1 promotes glycolysis by accelerate rearrangement of the cytoskeleton in tumor cells. (A) Relative glycolysis levels in GSN knockdown cells were measured by glucose uptake (n=3, one-way ANOVA) (left), lactate production (n=3, one-way ANOVA) (middle), and medium acidification (n=3, one-way ANOVA) (right). (B) Cells were transfected with empty vector, HBP1 or R378A, followed by glucose uptake assays (n=3, one-way ANOVA) (left) and lactate production assays (n=3, one-way ANOVA) (right). (C) HeLa cells stably expressing HBP1, R378A, HBP1+shGSN-1, R378A+shGSN-1, HBP1+shGSN-2, and R378A+shGSN-2 individually, the relative glycolysis levels in GSN knockdown cells were measured by glucose uptake (n=3, one-way ANOVA) (left) and lactate production assays (n=3, one-way ANOVA) (right). Date were the mean±SD. *p < 0.05, **p < 0.01.

Figure S5. High GSN expression predicts better overall survival in cervical cancer. Data from the GEPIA databases (<http://gepia.cancer-pku.cn/>). Kaplan-Meier survival analysis was used. Log-rank p values were shown.

Figure S6. The methylation of HBP1 at R378 alleviates its effects on expression of DNMT1, p53, p21 and p16 proteins. The protein levels of DNMT1, p53, p21 and p16 were verified by western blotting in HeLa and MGC803 cells with HBP1/R378A overexpression.
