# Supplementary figures and images for: Methylation of HBP1 by PRMT1 promotes tumor progression by regulating actin cytoskeleton remodeling
Source: Oncogenesis. 2022 Aug 8;11(1):45. doi: 10.1038/s41389-022-00421-7 (PMC9360041; doi:10.1038/s41389-022-00421-7)

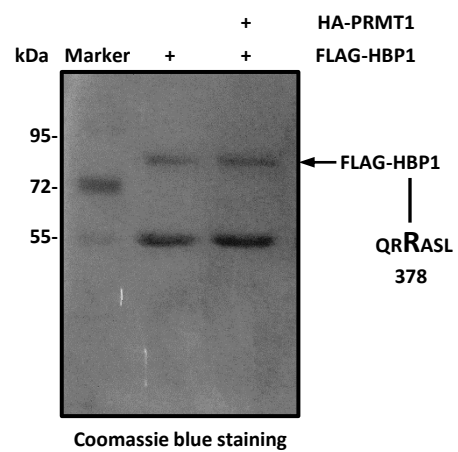

Figure S1

Supplement: Supplementary file 2 — Figure S1 [file 41389_2022_421_MOESM2_ESM.pdf]

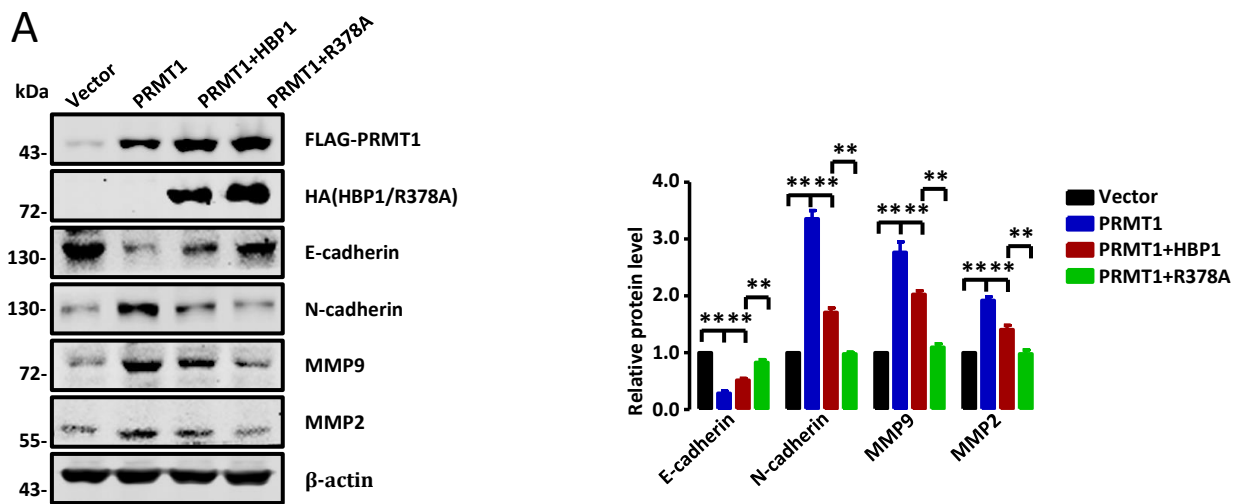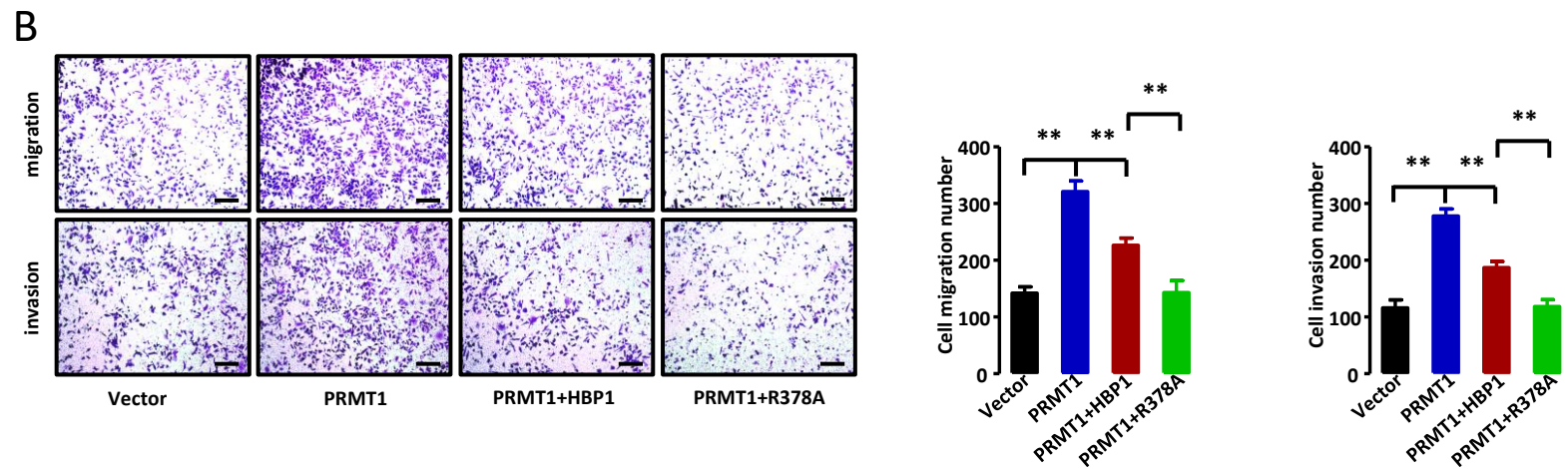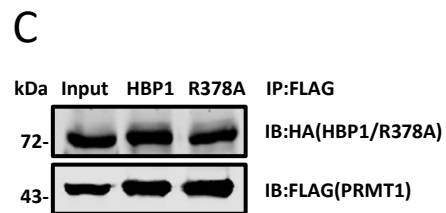

Figure S2

Supplement: Supplementary file 3 — Figure S2 [file 41389_2022_421_MOESM3_ESM.pdf]

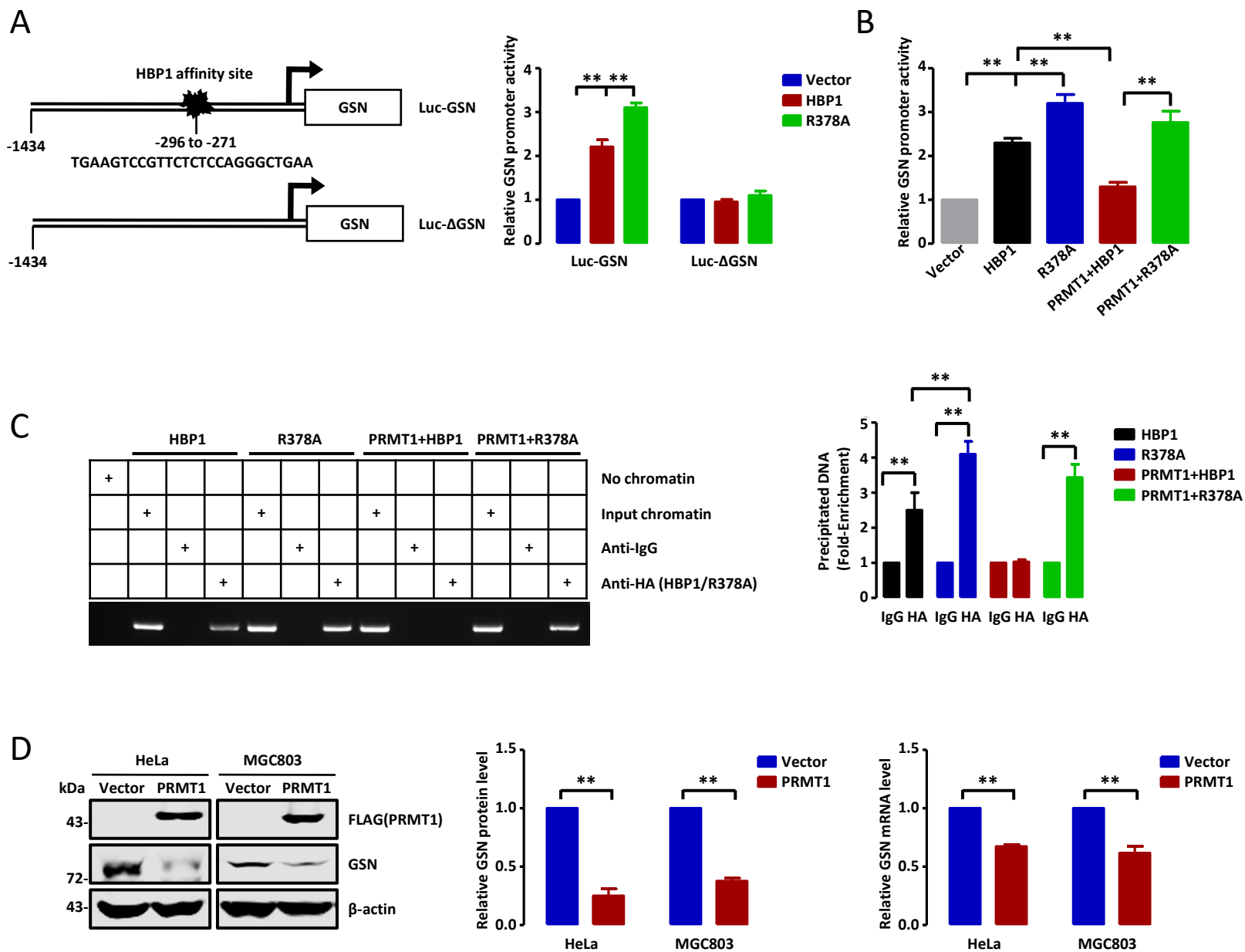

Figure S3

Supplement: Supplementary file 4 — Figure S3 [file 41389_2022_421_MOESM4_ESM.pdf]

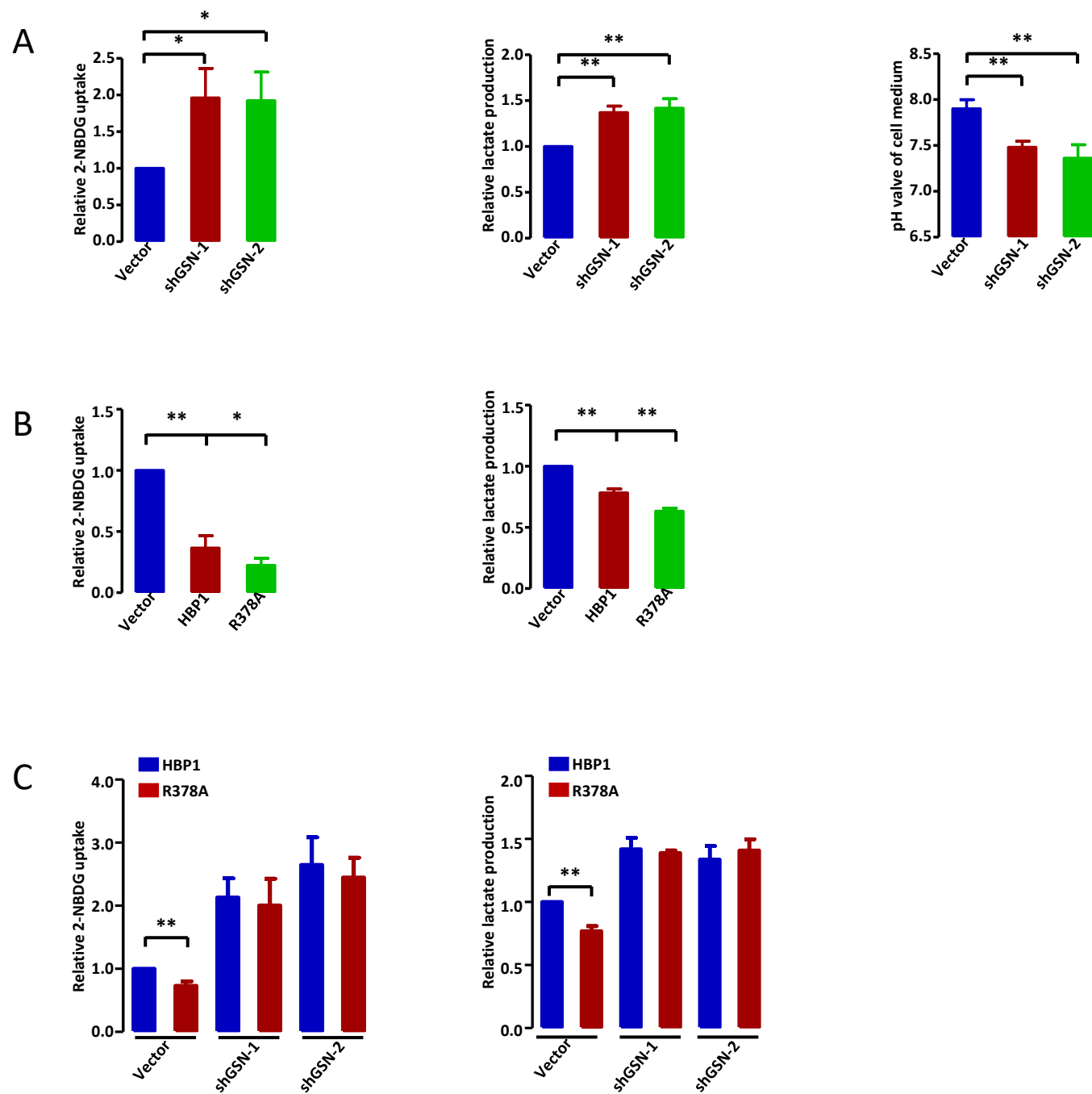

Figure S4

Supplement: Supplementary file 5 — Figure S4 [file 41389_2022_421_MOESM5_ESM.pdf]

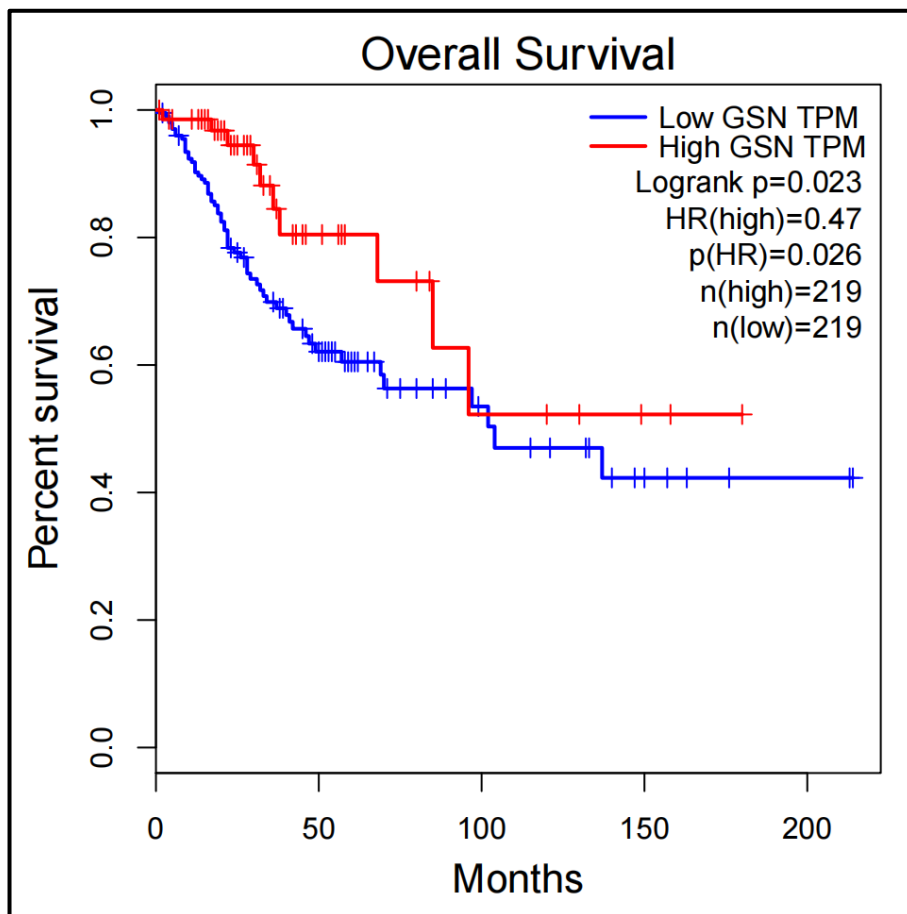

Figure S5

Supplement: Supplementary file 6 — Figure S5 [file 41389_2022_421_MOESM6_ESM.pdf]

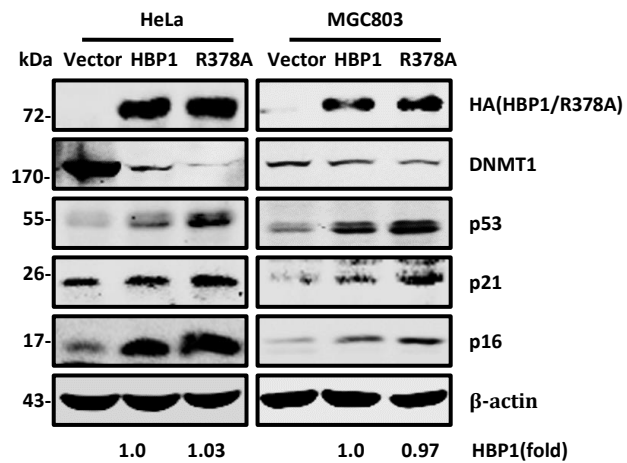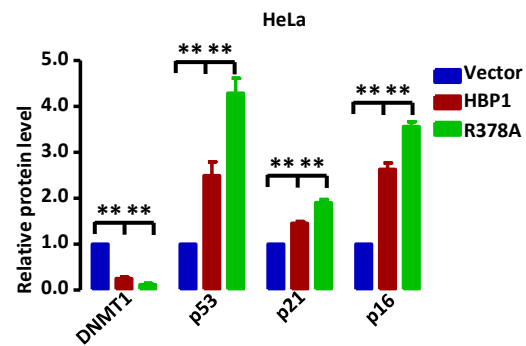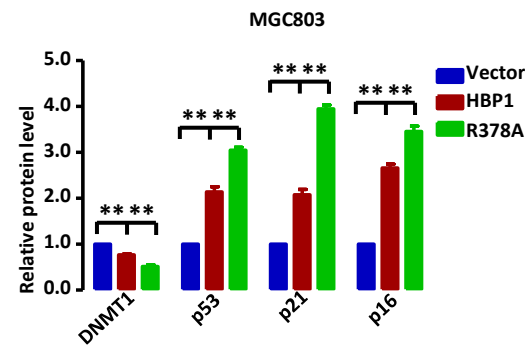

Figure S6

Supplement: Supplementary file 7 — Figure S6 [file 41389_2022_421_MOESM7_ESM.pdf]
